# Accurate height and length estimation in hospitalized children not fulfilling WHO criteria for standard measurement: a multicenter prospective study

**DOI:** 10.1007/s00431-024-05692-3

**Published:** 2024-07-25

**Authors:** Carole Ford Chessel, Julien Berthiller, Isabelle Haran, Lyvonne N. Tume, Christelle Bourgeaud, Michael Tsapis, Benedicte Gaillard-Le Roux, Evelyne Gauvard, Claire Loire, Camille Guillot, Karine Mouneydier, Paul Nolent, Thibault Blache, Fleur Cour Andlauer, Shancy Rooze, Corinne Jotterand Chaparro, Claire Morice, Fabien Subtil, Margaux Huot, Frédéric V Valla

**Affiliations:** 1 Pediatric Dietetic Unit, Hôpital Femme Mère Enfant, Hospices Civils de Lyon, 59 Bd Pinel, 69500 Lyon-Bron, France; 2 Public Health Department, Clinical Epidemiology and Research Unit, Hospices Civils de Lyon, 59 Bd Pinel, 69500 Lyon-Bron, France; 3 Pediatric Dietetic Unit, Hôpitaux Universitaires de Strasbourg, 1 Avenue Molière, 67000 Strasbourg, France; 4 Edge Hill University, St Helens Road, Ormskirk, Lancashire L39 4QP UK; 5 Pediatric Dietetic Unit, Hôpital de La Timone, Assistance Publique Des Hôpitaux de Marseille, 264 Rue Saint-Pierre, 13005 Marseille, France; 6 Pediatric Intensive Care Unit, Assistance Publique Des Hôpitaux de Marseille, 264 Rue Saint Pierre, 13385 Cedex 05 Marseille, France; 7 Pediatric Intensive Care Unit, Hôpital Femme-Mère-Enfant, Nantes University Hospital, Nantes, France; 8 Clinical Investigation Center, CIC INSERM 1413, Nantes University Hospital, Nantes, France; 9 Pediatric Intensive Care Unit, Hôpital Jeanne de Flandre, CHU Lille, Avenue Eugène Avinée, 59000 Lille, France; 10 Pediatric Dietetic Department, CHU Bordeaux, Place Amélie Raba-Léon, 33076 Bordeaux Cedex, France; 11 Pediatric Intensive Care Unit, CHU Bordeaux, Place Amélie Raba-Léon, 33076 Bordeaux Cedex, France; 12 Pediatric Cardiac Intensive Care Unit, Hôpital Louis Pradel, Hospices Civils de Lyon, 59 Bd Pinel, 69500 Lyon-Bron, France; 13 Pediatric Intensive Care, Hôpital Femme Mère Enfant, Hospices Civils de Lyon, 59 Bd Pinel, 69500 Lyon-Bron, France; 14 EA 7426 Joint Research Unit HCL-bioMérieux, 69003 Lyon, France; 15 Pediatric Intensive Care, Hôpital Universitaire Reine Fabiola, Avenue JJ Crocq 15, 1020 Laeken, Belgium; 16 Geneva School of Health Sciences, HES-SO University of Applied Sciences and Arts Western Switzerland, Geneva, Switzerland; 17 Pediatric Intensive Care Unit, University Hospital of Geneva, Rue Willy Donzé 6, 1205 Geneva, Switzerland; 18 Department of Biostatistics, UMR 5558, CNRS Université Claude Bernard Lyon 1, Hospices Civils de Lyon, Lyon, France

**Keywords:** Pediatrics, Anthropometry, Critical care, Nutritional status

## Abstract

In hospitalized children, height should be measured. When world health organization (WHO) height measurement gold standards is impossible, the ideal height estimation technique is still unclear. We conducted an international prospective study in eight different pediatric intensive care units to assess the accuracy, precision, practicability, safety, and inter-rater reliability of 12 different height estimation techniques, based on body segment measurement extrapolation, or other calculations using previous or projected heights. All extrapolation techniques were performed on each child, and later compared to their WHO gold standard heights. A total of 476 patients were enrolled. In the < 2-year subgroup, board length use and growth chart extrapolation performed best. In the ≥ 2-year subgroup, growth chart extrapolation and parents’ report were the most accurate, followed by height measurement alongside the body with a tape measure. In both groups, body segment extrapolations were poorly predictive and showed mean bias and limits of agreement that varied a lot with age. Most body segment-based techniques presented with frequent measurement difficulties, but children’s safety was rarely compromised. The inter-rater reliability of body segment measurement was low in the < 2-year subgroup.

*Conclusions*: To accurately estimate height in hospitalized children, health care professionals should integrate the accuracy, precision, practicability, and reliability of each measurement technique to select the most appropriate one. Body segment-based techniques were the least accurate and should probably not be used. Simple techniques like growth chart extrapolation, or measurement alongside the body (and length board measurement in the youngest) should be implemented in daily practice.

*Trial Registration*: The study protocol was registered (12^th^ April 2019) on the clinical-trial.gov website (NCT03913247).

**Supplementary Information:**

The online version contains supplementary material available at 10.1007/s00431-024-05692-3.

**Table:** 

**What is Known:** • *Height should be measured in hospitalized children to assess nutritional status and calculate various clinical parameters*. • *Many hospitalized children cannot be measured using WHO conventional height measurement methods. The ideal height estimation method has not been identified yet*.
**What is New:** • *Most estimation methods based on body segment measurement extrapolation fail to accurately predict height*. • *Board length use and growth chart extrapolation performed best in young children (≥2 years). Growth chart extrapolation and parents’ report were the most accurate, followed by height measurement alongside the body with a tape measure in older children*.

## Introduction

In hospitalized children, weight and height should be measured at admission, especially in pediatric intensive care units (PICU) [1, 2]. Children’s height evolves with age and requires monitoring as standard follow-up. Height allows for nutritional status assessment to calculate body mass index (BMI) and height-for-age *z* scores. It is also used to determine resting energy expenditure (Schofield equations), which is essential to prescribe adequate nutritional support and avoid over- and underfeeding which are associated with impaired outcomes [1, 2]. Height is necessary for the calculations of clinical parameters such as corporal surface area (CSA), which is important to prescribe some medications like chemotherapy, for burn scores, or for the interpretation of CSA indexed parameters like cardiac output. Height is also used to calculate ideal and adjusted weight in obese children, which is important to prescribe some medications with body composition distribution variation or to set mechanical ventilation parameters. Errors in height measurement will induce errors in all these parameters: for example, a 3.5% error would lead to a 2%, 7.5%, and 5% error respectively in CSA, BMI, and Schofield energy requirement calculations, which accuracies are already impacted by weight measurement errors (overhydration, indwelling devices, etc.) and by the 4% mean bias of Schofield equations [3]. Thus, reducing height measurement errors is important.

The world Health organization (WHO) has published gold standard procedures for children’s height and length measurement. In children below 2 years of age, length should be measured on a straight lying child using a length board. In children above 2 years of age, height is measured standing upright, using a height board [4]. In hospitalized children, however, these WHO conventional anthropometric approaches (“WHO gold standard”) for length and height measurement cannot always be undertaken. Some children cannot stand upright because of their clinical condition; others are equipped with tubes, drains, plasters, plaster casts, etc. that may interfere with height measurements or compromise their safety.

In children who cannot be measured following the WHO gold standards (e.g., cerebral palsy), other height estimation or extrapolation techniques have been proposed, including body segment length extrapolation or other estimation techniques [5–9]. In this study, we aim to assess, in hospitalized children, the accuracy, the reliability, and the safety of these techniques, as they have not been validated in this setting yet.

## Material and methods

We undertook a multicenter prospective cross-sectional study. It was conducted in PICU as we considered critically ill children would be the most likely to present with contra-indications to or difficulties to perform height measurement as per WHO gold standards (in relation to their severe critical condition and multiple equipment); we also hypothesized that the results could be extrapolated to less severe patients in pediatric wards.

Eight PICUs participated from Belgium, France, and Switzerland, from 2019 to 2022. Children (28 days to 18 years) were included if they did not fulfill the WHO gold standard criteria for height measurement at the time of enrolment (because of their clinical condition or any indwelling equipment). Each participant was later measured according to WHO criteria and acted as his own control to compare height estimation techniques to WHO gold standards measurements. The period between enrollment time point and WHO measurement timepoint had to be shorter than 5% expected natural height growth, based on WHO height and length velocity growth charts (supplemental digital content 1 provides these delays for various age ranges, based on WHO height velocity curves). Children with abnormal skeletal presentations (e.g., nanism, scoliosis, limb abnormalities) or retractions were excluded. Two groups of children were distinguished, based on their age (< 2 years, ≥ 2 years), as WHO criteria differ. Parental consent was obtained. Ethical clearance was obtained from the “OUEST III protection of persons” committee (7^th^ October 2019). The study protocol was registered (12^th^ April 2019) on the clinical-trial.gov website (NCT03913247).

During the child’s PICU stay, height estimation techniques were performed for each child:

- Extrapolation from length measurements of ulna, tibia, knee-heel, and half of the arm span (based on Chumlea and Gauld-Stevenson formulas) [6–8]
- Estimation from length measurement alongside the recumbent body with a tape measure and from the sum of body segment lengths (head + trunk + lower limbs)
- Estimation from a length board in children < 2 years (in cases where indwelling device or clinical condition may not allow fulfilling WHO gold standard criteria entirely and compromise its accuracy)
- Extrapolation from previous measurements allowing for height growth chart projection, from a hypothetical identical height for age *z*-score to the actual or most recent weight for age *z*-score, and from genetic parental height target
- Estimation from parents’ report
- Last height found in the child personal medical file or health records

All techniques are described in detail in Supplemental Material 2. Local investigators followed a 1-day in-person training to perform measurements following a written protocol, provided by the principal investigator to optimize measurement reproducibility. Ulna, tibia, arm span, and knee-heel measurements were performed with both a tape measure and a standard caliper (Cescorf Large Bone Anthropometer©, Nutriactiva, Mineapolis, USA). Measurements were performed by a trained local investigator, with the help of the nurse in charge of the patient.

When the child recovered and fulfilled WHO criteria, height was measured as per the WHO gold standard and each child served as his own control. Our main objective was to compare estimation techniques to the WHO gold standard in terms of “overall accuracy” or bias (defined as a mean relative error < 3.5%). We also considered the "individual accuracy" or precision of the techniques based on the percentage of patients presenting with a relative error < 3.5%, its variation with children’s height (considered a surrogate of children’s age), the dispersion of measurements, if these were 0-centered, and if numerous outliers were encountered.

We also aimed to assess each measurement technique safety (based on the number of patients presenting with clinical condition worsening during measurements or indwelling device accidental removal) and applicability (based on the number of patients presenting with impossible or difficult measurements such as reading result difficulty, child’s position holding difficulty, indwelling devices being obstacle to measurements, body segments difficulty to locate).

We further aimed to assess the inter-rater reliability. In two of the eight centers, all measurements were performed twice on the same child, by two different trained operators, blind to each other, and results were compared.

Patient characteristics and indwelling devices were collected to describe the population.

This study was reported using STARD, the most appropriate EQUATOR reporting checklist [10].

### Statistical analysis

A 3.5% relative error in height measurement was considered to significantly impact on CSA, BMI, and Schofield equation accuracy. The standard deviation of the relative error was expected to be equal to 4%, assuming 10% of children for whom either a technique or the WHO gold standard would not be available; thus, the inclusion of 231 children per group (< and ≥ 2 years) would provide a precision in the mean relative error estimate of ± 0.5% (mid-width of the 95% confidence interval). The absolute mean relative error was tested for each technique against 3.5% with a one-sided normal test. The percentage of children for whom the relative error was < 3.5% was described. The mean relative bias was estimated with its associated 95% confidence interval, and the Bland and Altman concordance plot was provided for each method. The intraclass correlation coefficient was calculated for each method to assess the inter-rater reliability. The percentage of children with a least one difficulty, and at least one safety issue was calculated per technique.

The accuracy analysis was performed on children with WHO gold standard measured within the acceptable time period defined previously; the safety and practicability analysis were performed on all the children for whom the measurements were attempted and the reliability analysis on those who were measured twice regardless their WHO gold standard measurement. Missing data were not imputed, and analysis was performed on available data. R software version 4.1.1 was used. A *p*-value less than 0.05 was considered significant.

## Results

### Patient characteristics

In total, 476 patients were enrolled (244 and 232 in the < 2-year and ≥ 2-year age subgroups respectively). Of them, 239 and 223 in each respective group could have their height measured as per WHO criteria and were further analyzed. In each age subgroup, 244 and 232, and 47 and 72, were analyzed regarding the safety-practicability and interrater reliability of the measurement respectively (see patient flow chart in Supplemental Material 3).

Table 1 (and Supplemental Material 3) presents patients’ characteristics and their indwelling devices or condition that compromised their height measurement. All children were equipped with at least one device and 73% were mechanically ventilated. Tables 2 and 3 present the percentages of patients who could have their height estimated for each technique: those were high (> 95%) except for parent’s report, and missing data were rare.

**Table 1:** Patients’ characteristics

Age group (years)	< 2 years *N* = 244	≥ 2 years *N* = 232	Total *N* = 476
Male gender	127 (52.0%)	134 (57.8%)	261 (54.8%)
Age (month)	5.0 (2.0–11.0)	99.00 (51.0–165.5)	21.00 (4.9–93.2)
Weight (kg)	6.2 (4.4–8.3)	25.5 (16.0–48.7)	11.30 (6.2–25.0)
PELOD2 severity score	5.0 (2.7–8.0)	4.0 (2.0–7.0)	4.5 (2.0–8.0)
Surgical patient	106 (43.4%)	114 (49.1%)	220 (46.2)
Invasive ventilation	113 (46.3%)	84 (36.2%)	197 (41.4%)
Non-invasive ventilation	102 (41.8%)	51 (22.0%)	153 (32.1%)
Sedated	114 (46.7%)	92 (39.7%)	206 (43.3%)
Indwelling catheter (venous or arterial)	219 (89.8%)	218 (94.4%)	437 (92%)
Indwelling urinary catheter	144 (59.0%)	171 (73.7%)	315 (66.2%)
Indwelling endotracheal tube	112 (45.9%)	81 (34.9%)	193 (40.5%)
Indwelling drains	89 (36.5%)	110 (47.4%)	199 (41.8%)
Indwelling gastric tube	181 (74.2%)	80 (34.5%)	261 (54.8%)
Indwelling stoma	4 (1.6%)	2 (0.9%)	6 (1.3%)
Indwelling intracranial pressure catheter	6 (2.5%)	11 (4.8%)	17 (3.6%)
Indwelling regional analgesia catheter	8 (3.3%)	14 (6.1%)	22 (4.6%)
Head dressing	10 (4.1%)	17 (7.4%)	27 (5.7%)
Other large dressings	109 (44.7%)	106 (45.9%)	215 (45.3%)
Ongoing renal replacement therapy	15 (6.0%)	21 (9.0%)	36 (7.6%)
Ongoing extra corporeal life support	2 (0.8%)	4 (1.7%)	6 (1.3%)
Casts/corset, braces, and splints/cervical collar/traction	5 (2.0%)	9 (3.9%)	14 (2.9%)

Results are presented in median (IQR 25–75) or number (percentage). *PELOD* pediatric logistic organ dysfunction score 2, *ICP* intracranial pressure

**Table 2:** Children < 2 years. Concordance, percentage of absolute value of the relative error, number of difficulties or safety issues, and intraclass correlation coefficients of each extrapolation or estimation of height or length methods

Length extrapolation or estimation method (*N*-%^a^)	Mean bias (cm) [95% CI]	*p*-value (relative error ≠ 3.5%)	% of children with relative error < 3.5%	Difficulties (*N* and %^b^)	Safety issue (*N* and %^b^)	Interrater reliability ICC [95% CI]^b^
Tibia tape measure (239–100%)	3.6 [3.0; 4.3]	1.000	72 (30.1)	131 (53.7%)	0 (0.0%)	0.92 [0.85; 0.95]
Tibia caliper (239–100%)	3.2 [2.5; 3.8]	1.000	79 (33.0)	156 (63.9%)	3 (1.2%)	0.93 [0.88; 0.96]
Knee-Heel tape measure Gauld (239–100%)	− 0.4 [− 0.9; 0.1]	< 0.001	124 (51.9)	7 (2.9%)	0 (0.0%)	0.96 [0.93; 0.98]
Knee-Heel caliper Gauld (239–100%)	− 2.1 [− 2.6; − 1.6]	0.013	96 (40.2)	16 (6.6%)	3 (1.2%)	0.97 [0.95; 0.99]
Knee-Heel tape meas. Chumlea (239–100%)	14.7 [14.1; 15.3]	1.000	5 (2.1)	7 (2.9%)	0 (0.0%)	0.96 [0.93; 0.98]
Knee-Heel caliper Chumlea (239–100%)	13.2 [12.6; 13.8]	1.000	5 (2.1)	16 (6.6%)	3 (1.2%)	0.97 [0.95; 0.99]
Ulna tape measure (239–100%)	8.4 [7.7; 9.0]	1.000	19 (7.8)	71 (29.2%)	0 (0.0%)	0.96 [0.94; 0.98]
Ulna caliper (238–99.6%)	8.1 [7.5; 8.8]	1.000	21 (8.8)	129 (53.1%)	2 (0.8%)	0.95 [0.91; 0.97]
Half of the arm span (237–99.2%)	8.9 [8.1; 9.6]	1.000	24 (10.0)	64 (26.3%)	2 (0.8%)	0.96 [0.93; 0.98]
Sum of body segments (239–100%)	0.5 [0.2; 0.9]	< 0.001	155 (64.8)			0.96 [0.93; 0.98]
Head				26 (10.7%)	0 (0.0%)	0.78 [0.63; 0.87]
Trunk				116 (47.5%)	0 (0.0%)	0.73 [0.56; 0.84]
Lower limb				119 (48.8%)	1 (0.4%)	0.92 [0.87; 0.96]
Alongside the body tape measure (233–97.5%)	− 0.02 [− 0.3; 0.3]	< 0.001	182 (78.1)	29 (11.9%)	1 (0.4%)	0.99 [0.98; 0.99]
Length board (232–97.1%)	− 0.3 [− 0.5; 0.0]	< 0.001	200 (86.2)	30 (12.3%)	0 (0.0%)	0.99 [0.99; 1.00]
Growth chart extrapol. (225–94.1%)	− 0.1 [− 0.4; 0.1]	< 0.001	189 (84.0)			
Weight for age *z*-score extrapol. (238–99.6%)	− 1.0 [− 1.4; − 0.6]	< 0.001	121 (50.8)			
Genetic target extrapol. (229–95.8%)	2.0 [1.5; 2.6]	0.567	112 (48.9)			
Parents’ report (189–79.1%)	− 0.7 [− 1.1; − 0.4]	< 0.001	136 (72.0)			
Health record/medical files (229–95.8%)	− 1.6 [− 1.9; − 1.3]	< 0.001	158 (69.0)			

^a^Number of patients (and its percentage of the 239 included patients) who could be measured according to the WHO gold standard and allow for comparison to each technique (these figures were used for mean bias, *p*-value, and % of children with relative error < 3.5%)

^b^% based on all the children who were assessed by the estimation technique, regardless of the WHO comparability

**Table 3:** Children ≥ 2 years. Concordance, percentage of absolute value of the relative error, number of difficulties or safety issues, and intraclass correlation coefficients of each extrapolation or estimation of height or length methods

Height extrapolation or estimation method (*N*-%^a^)	Mean bias (cm) [95% CI]	*p*-value (relative error ≠ 3.5%)	% of children with relative error < 3.5%	Difficulties (*N* and %^b^)	Safety issue (*N* and %^b^)	Interrater reliability ICC^b^
Tibia tape measure (223–100%)	− 0.5 [− 1.4; 0.3]	< 0.001	145 (65.0)	37 (15.9%)	0 (0.0%)	0.99 [0.98; 0.99]
Tibia caliper (223–100%)	− 1.4 [− 2.3; − 0.6]	< 0.001	145 (65.0)	45 (19.4%)	0 (0.0%)	0.98 [0.97; 0.99]
Knee-Heel tape measure Gauld (223–100%)	0.0 [− 0.6; 0.7]	< 0.001	149 (66.8)	15 (6.5%)	0 (0.0%)	0.99 [0.99; 1]
Knee-Heel caliper Gauld (223–100%)	− 1.3 [− 2.0; − 0.6]	< 0.001	143 (64.1)	19 (8.2%)	0 (0.0%)	1 [0.99; 1]
Knee-Heel tape meas. Chumlea (223–100%)	− 1.2 [− 2.1; − 0.3]	< 0.001	91 (40.8)	15 (6.5%)	0 (0.0%)	0.99 [0.99; 1]
Knee-Heel caliper Chumlea (223–100%)	− 2.4 [− 3.3; − 1.4]	< 0.001	89 (39.9)	19 (8.2%)	0 (0.0%)	1 [0.99; 1]
Ulna tape measure (222–99.6%)	1.5 [0.7; 2.3]	< 0.001	131 (59.0)	19 (8.2%)	0 (0.0%)	0.99 [0.98; 0.99]
Ulna caliper (222–99.6%)	0.9 [0.2; 1.7]	< 0.001	138 (62.2)	28 (12.5%)	1 (0.4%)	0.98 [0.97; 0.99]
Half of the arm span (220–98.7%)	1.3 [0.8; 1.9]	< 0.001	147 (66.8)	26 (11.2%)	0 (0.0%)	1 [0.99; 1]
Sum of body segments (223–100%)	2.0 [1.4; 2.6]	< 0.001	163 (73.1)			0.98 [0.96; 0.99]
Head				34 (14.7%)	0 (0.0%)	0.75 [0.62; 0.83]
Trunk				140 (60.3%)	0 (0.0%)	0.78 [0.67; 0.86]
Lower limb				143 (61.6%)	0 (0.0%)	0.97 [0.96; 0.98]
Alongside the body tape measure (211–94.6%)	1.9 [1.4; 2.4]	–	160 (75.8)	62 (26.7%)	0 (0.0%)	0.99 [0.99; 1]
Growth chart extrapol. (212–95.1%)	− 0.7 [− 1.7; 0.3]	< 0.001	193 (91.0)			
Weight for age *z*-score extrapol. (223–100%)	0.5 [− 0.5; 1.4]	< 0.001	129 (57.8)			
Genetic target extrapol. (206–92.4%)	3.2 [1.4; 4.9]	0.214	111 (53.9)			
Parents’ report (182–81.6%)	− 0.42 [− 0.9; 0.0]	< 0.001	162 (89.0)			
Health records/Medical files (213–95.6%)	− 4.89 [− 6.0; − 3.7]	0.599	147 (69.0)			

^a^Number of patients (and its percentage of the 223 included patients) who could be measured according to the WHO gold standard and allow for comparison to each technique (these figures were used for mean bias, *p*-value, and % of children with relative error < 3.5%)

^b^% based on all the children who were assessed by the estimation technique, regardless of the WHO comparability

### Accuracy

**Fig. 1:**
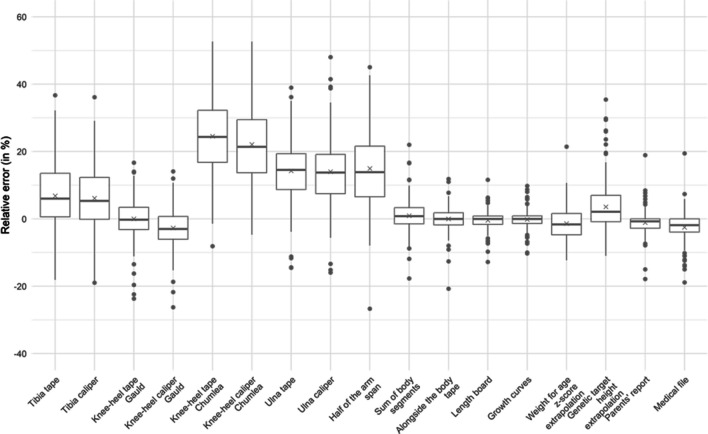
Boxplot of height relative errors compared to WHO gold standard, in the < 2-year subgroup

**Fig. 2:**
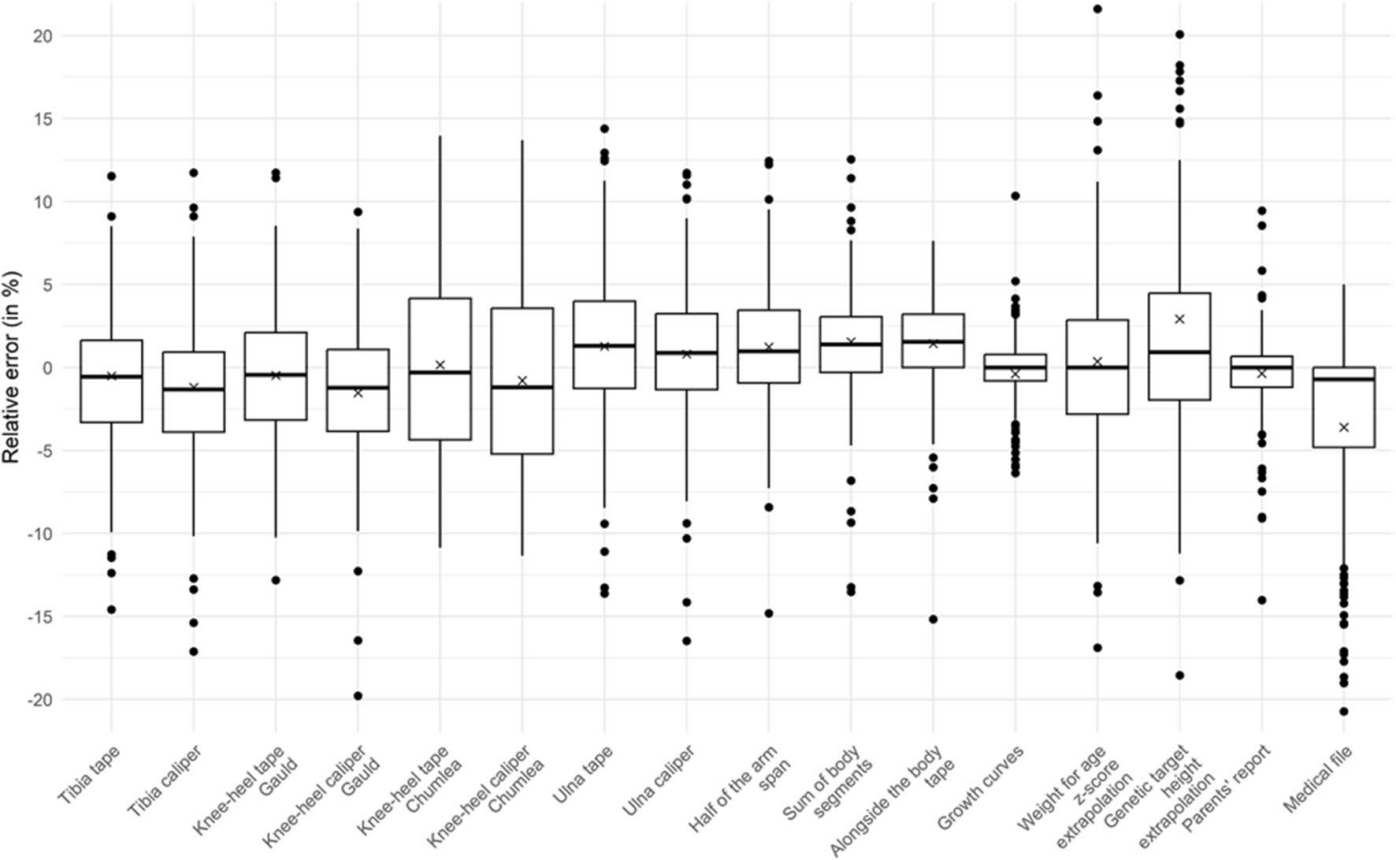
Boxplot of height relative errors compared to WHO gold standard, in the ≥ 2-year subgroup

**Fig. 3:**
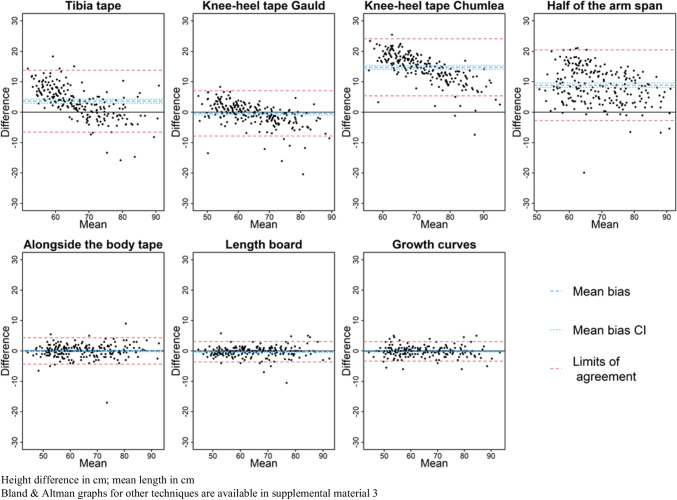
Bland and Altman graphs, by method and length (surrogate of age) in children < 2 years

**Fig. 4:**
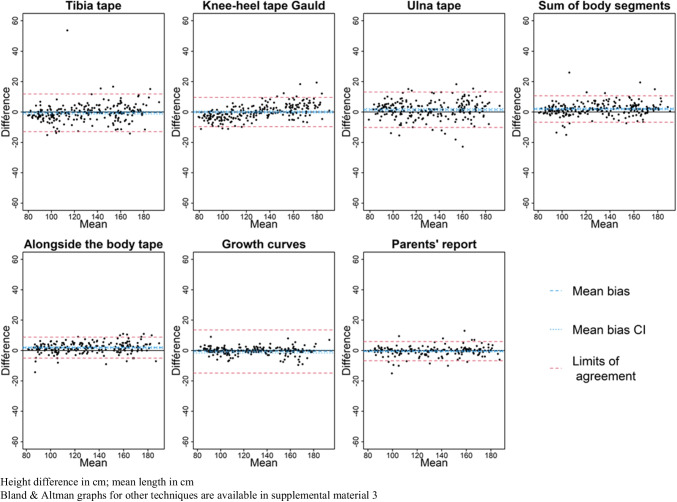
Bland and Altman graphs, by method and height (surrogate of age) in children ≥ 2 years

Figures 1 and 2 present the relative error (%) of each estimation technique compared to the WHO gold standard. Figures 3 and 4 present the mean bias and limits of agreement variations with height of 7 key techniques (results of all techniques are shown in Supplemental Material 3). Table 2 and 3 present the mean bias and the relative error compared to the WHO gold standard of each technique assessed to estimate height (detailed results: in Supplemental Material 3 and 4). In the < 2-year subgroup, body segment length extrapolations were poorly predictive of patients’ length, and board length (− 0.3 cm [95% CI − 0.5; 0.0]) and growth chart extrapolation (− 0.1 cm [95% CI − 0.4; 0.1]) performed best. In the ≥ 2-year subgroup, body segment length extrapolations were better than in younger children but remained poorly accurate, and growth chart extrapolation (− 0.7 cm [95% CI − 1.7; 0.3]) and parents’ report (− 0.42 cm [95% CI − 0.9; 0.0]) were the most accurate. However, the mean bias and the limits of agreement of each technique varied with age (height being a surrogate of age) as shown in their Bland and Altman graphs presented in Figs. 3 and 4.

As a majority (64.6%) of the patients were enrolled from two centers, a sensitivity analysis was conducted to describe the difference with the other centers. Compared to the WHO gold standard, the accuracy of the estimation techniques was higher in these two centers (Supplemental Digital Content 5).

### Safety and feasibility

Safety issues and difficulties encountered during measurements are presented in Tables 2 and 3 (and in Supplemental Material 3 and 4). Safety issues (almost all in relation with caliper use) were rarely encountered and were not severe. Difficulties were more frequently encountered in the youngest (*p* = 0.03): in the < 2-year subgroup, tibia measurement was the most difficult to perform, followed by body segments and ulna measurement (tibial lateral condyle and ulna styloid process locating difficulty was the most frequently reported). In the ≥ 2-year subgroup, length measurements alongside the body and body segment measurements were the most difficult to perform followed by tibia length. Caliper use also generated more difficulties than tape measure use in both groups (*p* ≤ 0.01). Measurements caused pain in a few children and the caliper frightened some of them. Difficulties to position the patient and for them to maintain the position during measurements were more frequent for the half of the arm span but rare for other measurements. Finally, locating body segments and tibia and ulna extremities were the main cause of the reported difficulties.

### Inter-rater reliability

In the inter-rater reliability analysis, 119 patients were enrolled. The inter-rater reliability is presented in Tables 2 and 3 (and in Supplemental Material 3 and 4). Body segment measurements had the lowest inter-rater reliability. In the < 2-year subgroup, tibia and ulna measurements were the less reliable techniques, and in the ≥ 2-year subgroup, all measures had high intraclass correlation coefficients.

**Fig. 5:**
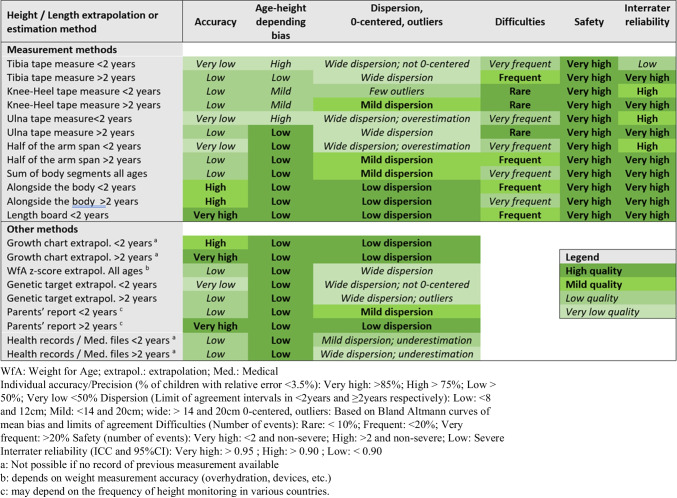
Comparison of overall quality of all methods of height or length extrapolation, based on their accuracy, safety and reliability

Figure 5 presents a summary of each technique accuracy, safety, difficulty, and inter-rater reliability, in order to help clinicians appropriately select the best technique to estimate height.

## Discussion

This is the first study that has tested several techniques to estimate height or length when the WHO conventional anthropometric approaches could not be performed. They presented with a large variation in their accuracy, precision, practicability, and inter-rater reliability, but were all safe. Body segment-based techniques are the least accurate and should not be used, and simple techniques like growth chart extrapolation or measurement alongside the body and length board measurement in the youngest were the most useful.

Systematic measurement of height is good practice in hospitalized children [11]. However, limited reports are available in the literature to assess how this is done in children not fulfilling WHO criteria, such as PICU patients, and from any pediatric ward. In a study conducted in PICU, height measurement increased from 32 to 99% of the patients after a training program and the implementation of ulna and tibia length extrapolation [12]. However, the accuracy of such techniques remained questionable.

Body segment measurements to extrapolate height have been validated in specific children populations (e.g., neuromuscular weakness, cerebral palsy). Their accuracy and inter-rater reliability were tested in school age healthy Australian children (5 to 19 years old) by Gauld et al. and showed a satisfactory overall accuracy relevant for research purposes; but at an individual level, they may present with a lack of precision which limits their use in clinical practice [7, 8]. Other studies found heterogenous accuracies of these techniques [13, 14] and a recent review concluded that most studies showed no correspondence between extrapolated and real height [9]. Not surprisingly, in our youngest subgroup of children, these techniques were of overall poor quality, as most of the extrapolation calculation formulas were developed in children above 5 years of age. However, in the older subgroup, their precision remained low.

We found inter-rater reliability was high in most techniques, after a short training period of the local investigators. The safety of all measurements was very high even in critically ill children. A lot of difficulties were encountered, which was expected in PICU. However, most measurement were possible with few missing data. The use of a dedicated caliper did not increase measurement accuracy (compared to a simple inexpensive tape measure as shown by Spender et al. in 1989 [15]), and generated more difficulties, and we do not recommend its use.

The precision, safety, reliability, and practicability of the technique should be taken into consideration when choosing which one to implement in daily clinical practice. Accuracy was sometimes satisfactory in the overall subgroup, but with large variations within the children’s ages, which compromises its precision at the individual level. We pragmatically presented in Fig. 5a qualitative summary of the features of all techniques assessed, to allow selecting the optimal one to implement in clinical practice. In the youngest subgroup, length board measurement was considered a high-quality technique and was possible in most cases (expect in those with scalp catheter or head dressings). Measurement alongside the body and growth chart extrapolation also performed well. In the older subgroup, measurement alongside the body, growth chart extrapolation, and parental report achieved the highest overall quality. However, if no previous measurement is available or in countries where regular height measurement is not standard of care, growth chart extrapolation and parental report may be impossible.

Ulijaszek et al. [16] reviewed anthropometric measurement errors in children, finding that weight and height measurements were accurate and reproductible, but other anthropometric measurements were not (e.g., waist and hip circumferences, skinfolds). Some limb measurements used to extrapolate height or length in our study similarly presented poor inter-rater reliability as per Ulijaszek definition (*R* > 0.95) and thus failed to accurately predict height.

Outside the cerebral palsy setting, we found few studies that have assessed the reliability of height estimation techniques confirming our results. Scalercio et al. compared newborn length measurements using a length board and a tape measure and found that weighted kappa coefficient and intraclass correlation coefficient indicated good to excellent agreement, but no other techniques were tested [17]. In children with learning difficulties, Hardy et al. [18] found disappointing wide bland–Altman limits of agreement between measured supine length and that estimated from segmental measurements. Young et al. performed a systematic review [19] of weight estimation techniques in children (in both hospitalized and healthy children) and showed that parent’s estimation and length-based techniques predicted the most accurately children’s body weight (which may be difficult to measure), which was confirmed in a recent study conducted by O’Leary et al. [20]. Consequently, this is crucial to measure length accurately to correctly estimate weight in hospitalized children who cannot be weighed easily or accurately.

Our study was conducted in PICU. However, a lot of hospitalized children outside the PICU also have clinical conditions or indwelling devices impairing height measurement. The translation of these PICU results to the ward setting might thus be possible. The choice of a simple technique such as the measurement alongside the body with a tape measure appears the most useful and is easier than all other sophisticated techniques.

### Limitations

The interrater reliability of growth charts, WfA *z*-score, and genetic target extrapolation was not tested, but these techniques do not depend on anthropometric measurements and strictly following a written protocol should have limited the bias. However, this is the first study that has tested numerous techniques to estimate height in hospitalized children, using a standardized and homogeneous procedure for each of them to limit potential bias, and focusing on various key features, relevant to clinical practice (precision, practicability, safety, reliability). Children with limb abnormalities or abnormal skeletal presentations or significant retractions were excluded and results should not be extrapolated to this population. Two thirds of the measurements were performed by the same operator; however, his higher accuracy rate mainly reflects the impact of the operator’ experience, as homogenous training to measurements techniques was part of the study design.

## Conclusions

Numerous techniques have been proposed to estimate hospitalized children’s height when the WHO conventional anthropometric approaches are not applicable. Health care professionals should consider a number of factors (accuracy, precision, practicability, and reliability) in their selection of the ideal technique to use for height measurement in their specific practice settings. Body segment-based techniques are the least accurate and should probably not be used. Simple techniques like growth chart extrapolation or measurement alongside the body and length board measurement in the youngest are the most useful. New extrapolation equations have recently been proposed [21] but still require adequate validation prior to clinical implementation.

## Supplementary Information

Below is the link to the electronic supplementary material.

Supplementary file1 (DOCX 16 KB)

Supplementary file2 (DOC 8900 KB)

Supplementary file3 (DOCX 1326 KB)

Supplementary file4 (DOCX 28 KB)

Supplementary file5 (DOCX 18 KB)

## Data Availability

The data that support the findings of this study are not openly available due to reasons of sensitivity and are available from the corresponding author upon reasonable request. Data are located in controlled access data storage at Hospices Civils de Lyon.

## Abbreviations

BMI: Body mass index
CSA: Cross-sectional area
PICU: Pediatric intensive care unit
WHO: World Health Organization
