# Supplementary material for: Accurate height and length estimation in hospitalized children not fulfilling WHO criteria for standard measurement: a multicenter prospective study
Source: Eur J Pediatr. 2024 Jul 25;183(10):4275–86. doi: 10.1007/s00431-024-05692-3 (PMC11413069; doi:10.1007/s00431-024-05692-3)
Supplement: Supplementary file 1 — Supplementary file1 (DOCX 16 KB) [file 431_2024_5692_MOESM1_ESM.docx]

Supplemental table 1: Time corresponding to a 5% height or length natural increase, according to WHO height velocity charts.

| **Age ranges** | **Mean height/Length 5% gain** | **Number of days to achieve a mean height/length 5% gain** |
| --- | --- | --- |
| **0-3 months** | + 8 cm | 5 days |
| **3-6 months** | + 6 cm | 5 days |
| **6-12 months** | + 8 cm | 9 days |
| **1-2 years** | + 11cm | 18 days |
| **2-4 years** | + 16 cm | 37 days |
| **4- 10 years Girls** | + 35 cm | 110 days |
| **4- 12 years boys** | + 44 cm | 146 days |
| **10-18 years girls** | + 28 cm | 146 days |
| **12-18 years boys** | + 28 cm | 110 days |

We aimed to compare height extrapolation techniques (performed at the time when children couldn’t be measured according to WHO gold standard because of their severe clinical condition), to WHO gold standard when each child had recovered enough to be measured according to WHO gold standard. Each child served as his own control.

However, as natural growth may occur between these two timepoints, we have excluded children if WHO gold standard could not be performed in “reasonable” times, not to be biased by natural growth.

We considered that 5% natural growth would be significant to alter the comparison of the measures performed at the two timepoints.

However, in sick children, pathophysiological adaptation processes often stop height growth (children do not usually grow during critical illness), which may drastically reduce the risk of natural growth bias in the study cohort.

5% natural growth corresponds to different times depending on the child’s age, as growth velocity depends on age; the above table presents the 5% expected natural growth and the corresponding times by age ranges; these were applied in our study to exclude patients from analysis when Gold standard could not be performed in due times. (this happened only in 1 child of the <2 years group, but did not in the older group as time ranges were a lot longer).

**Supplemental table 2: Delay between first set of height extrapolation measurements and WHO gold standard measurement**

|  | < 2years subgroup | > 2years subgroup |
| --- | --- | --- |
| Delay (in days) | 4 (2-5) | 7 (4-12) |

Results are presented in medians (IQR 25-75)
