# Supplementary material for: Accurate height and length estimation in hospitalized children not fulfilling WHO criteria for standard measurement: a multicenter prospective study
Source: Eur J Pediatr. 2024 Jul 25;183(10):4275–86. doi: 10.1007/s00431-024-05692-3 (PMC11413069; doi:10.1007/s00431-024-05692-3)
Supplement: Supplementary file 2 — Supplementary file2 (DOC 8900 KB) [file 431_2024_5692_MOESM2_ESM.doc]

**Measurement methods**

**Estimation of length and height methods**

- Extrapolation from ulna length measurement (gauld 2004 Gauld 2003)
- Extrapolation from tibia length measurement (Gauld 2004)
- Extrapolation from half of the arm span measurement (gauld 2004)
- Extrapolation from knee heel measurement (gauld 2004) based on Chumlea and Gauld formulas.
- Estimation from length measurement alongside the recumbent body with a tape measure
- Estimation from the sum of body segment length measurements alongside the recumbent body (head + trunk + lower limbs) with a tape measure
- Estimation from a length board in children <2 years
- Extrapolation from previous measurements allowing for height growth chart projection.
- Extrapolation from a hypothetical identical height for age z-score to the actual or most recent weight for age z-score
- Extrapolation from genetic parental height target
- Estimation from parents’ report
- Last Height found in the child’s health records / medical file

**Tibia length / Tape measure**

**Measurement:**

- Position the child in a recumbent position
- One operator holds the lower limb so that the thigh and the leg form a 90° angle.
- A second operator locates the knee medial joint space (tibia head) and the tibia distal medial malleolus.
- The second operator extends the tape measure between these two locations along the internal side of the leg.
- The second operator reads the measure in cm (+/- 0.5cm).

**Height extrapolation Formulas (Gauld et al.):**

Girls: **Height (cm) = 2,771 x T + 1,457 x age (in years) + 37,748**

Boys: **Height (cm) = 2,758 x T + 1,717 x age (in years) + 36,509**

*T = tibia length (cm)*


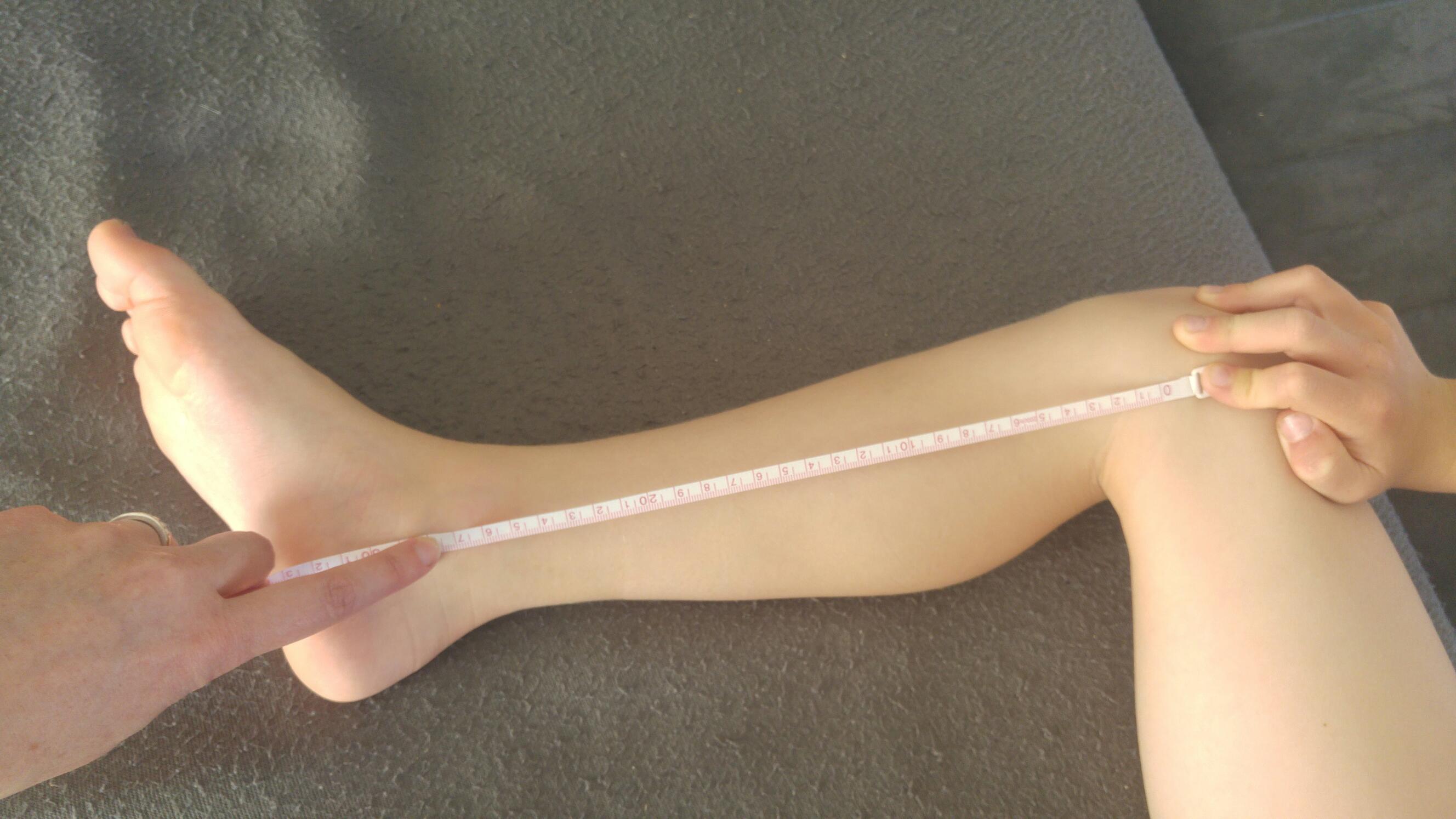


**Tibia length / Caliper**

**Measurement:**

- Position the child in a recumbent position.
- One operator holds the lower limb so that the thigh and the leg form a 90° angle.
- A second operator locates the knee medial joint space (tibia head) and the tibia distal medial malleolus
- The second operator places the fix part of the caliper towards the knee interline and moves the other end of the caliper towards the maleola, along the internal side of the leg
- The second operator reads the measure in cm +/- 0.1cm

**Height extrapolation Formulas (Gauld et al.):**

Girls: **Height (cm) = 2,771 x T + 1,457 x age (in years) + 37,748**

Boys: **Height (cm) = 2,758 x T + 1,717 x age (in years) + 36,509**

*T = tibia length (cm)*


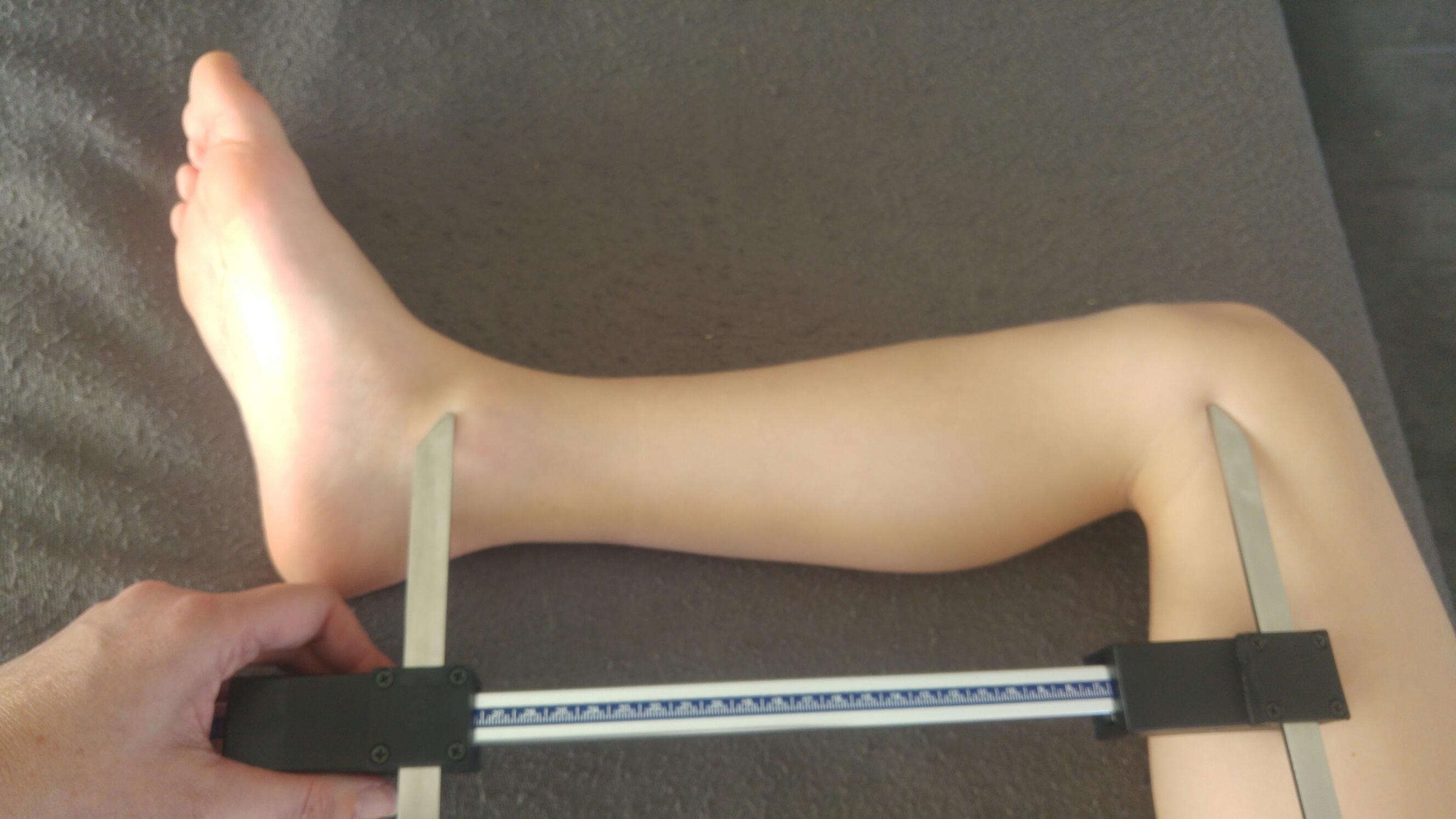


**Knee Heel length / tape measure**

**Measurement:**

- Position the child in a recumbent position.
- One operator holds the lower limb so that the thigh and the leg form a 90° angle.
- A second operator places the tape measure on the upper side of the knee, towards the heel, along the internal side of the leg.
- The second operator reads the measure in cm (+/- 0.5cm).

**Height extrapolation Formulas**

- Boys:

Chumlea formula:

Height (cm) = 40,54 + 2,22 x KH

Gauld-Stevenson formula:

Taille (cm) = 2.423 x KH + 1.327 x age (in years) + 21.818

- Girls:

Chumlea formula:

Height (cm) = 43,21 + 2,15 X KH

Gauld-Stevenson formula:

Height (cm) = 2.473 x KH + 1.187 x age (in years) + 21.151

*KH = Knee-Heel length (cm)*

*
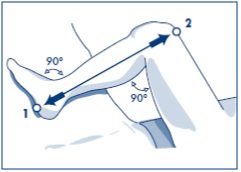
*

**Knee Heel length / caliper**

**Measurement:**

- Position the child in a recumbent position.
- One operator holds the lower limb so that the thigh and the leg form a 90° angle.
- A second operator places the caliper fix part towards the upper side of the knee, and moves the other end of the caliper towards the heel, along the internal side of the leg.
- The second operator reads the measure in cm (+/- 0.1cm).

**Height extrapolation Formulas**

- Boys:

Chumlea formula:

Height (cm) = 40,54 + 2,22 x KH

Gauld-Stevenson formula:

Taille (cm) = 2.423 x KH + 1.327 x age (in years) + 21.818

- Girls:

Chumlea formula:

Height (cm) = 43,21 + 2,15 X KH

Gauld-Stevenson formula:

Height (cm) = 2.473 x KH + 1.187 x age (in years) + 21.151

*KH = Knee-Heel length (cm)*

*
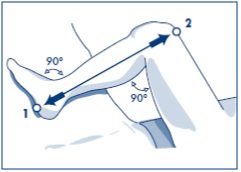
*

**Ulna length / Tape measure**

**Measurement:**

- Position the child in a recumbent position
- One operator holds the upper limb so that the arm and forearm form a 90° angle. The palm is positioned downwards, fingers are extended.
- A second operator locates the olecranon and the ulna distal styloid process
- The second operator extends the tape measure between these two locations along the forearm.
- The second operator reads the measure in cm (+/- 0.5cm).

**Height extrapolation Formulas (Gauld et al.):**

- Boys:

**Height (cm) = 4,605 x U + 1,308 x age (in years) + 28,003**

- Girls:

**Height (cm) = 4,459 x U + 1,315 x age (in years) + 31,485**

*U = Ulna length (cm)*


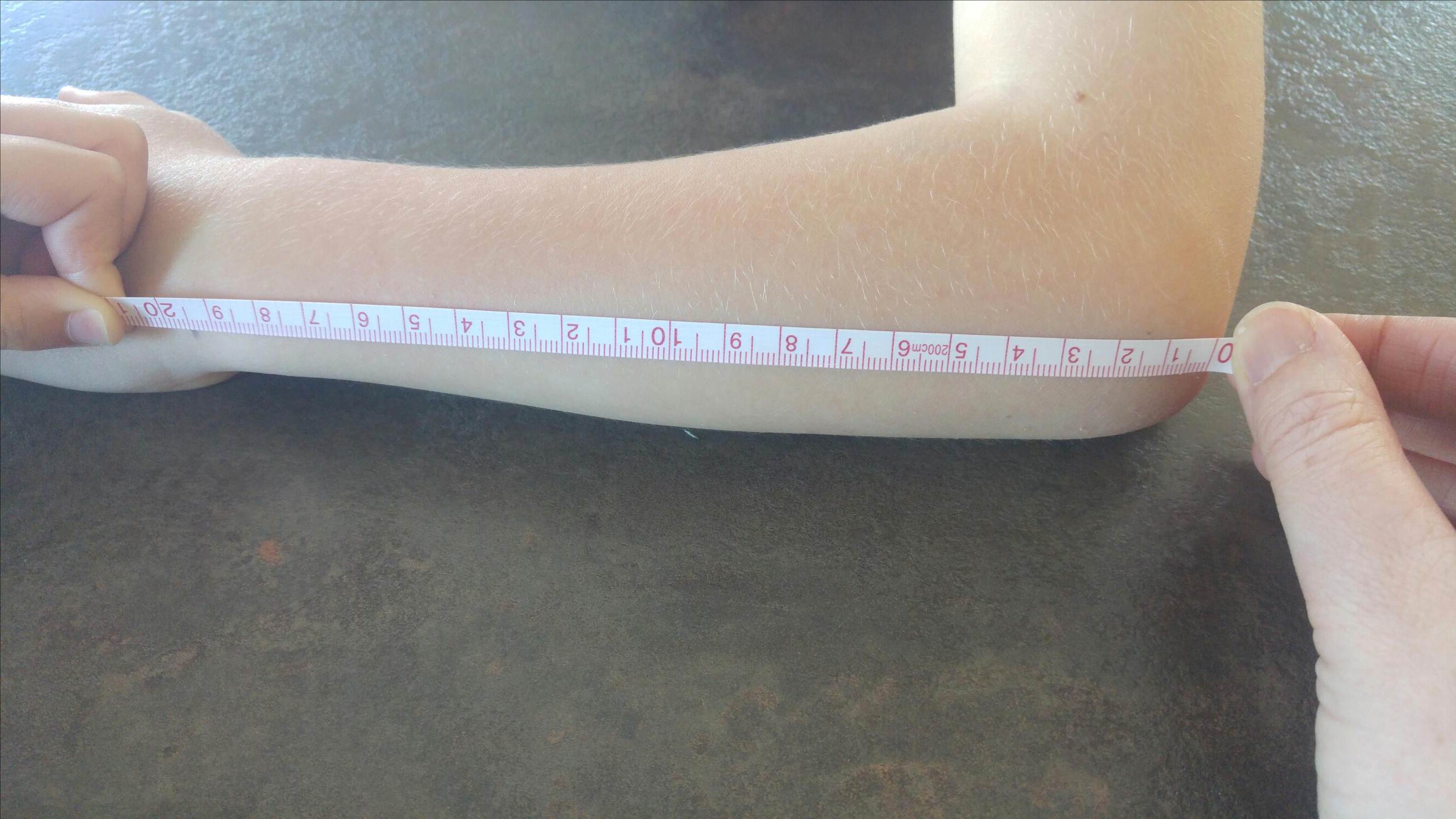


**Ulna length / Caliper**

**Measurement:**

- Position the child in a recumbent position.
- One operator holds the upper limb so that the arm and forearm form a 90° angle. The palm is positioned downwards, fingers are extended.
- A second operator locates the olecranon and the ulna distal styloid process.
- The second operator extends the caliper between these two locations along the forearm.
- The second operator reads the measure in cm (+/- 0.1cm).

**Height extrapolation Formulas (Gauld et al.):**

- Boys:

**Height (cm) = 4,605 x U + 1,308 x age (in years) + 28,003**

- Girls:

**Height (cm) = 4,459 x U + 1,315 x age (in years) + 31,485**

*U = Ulna length (cm)*

*
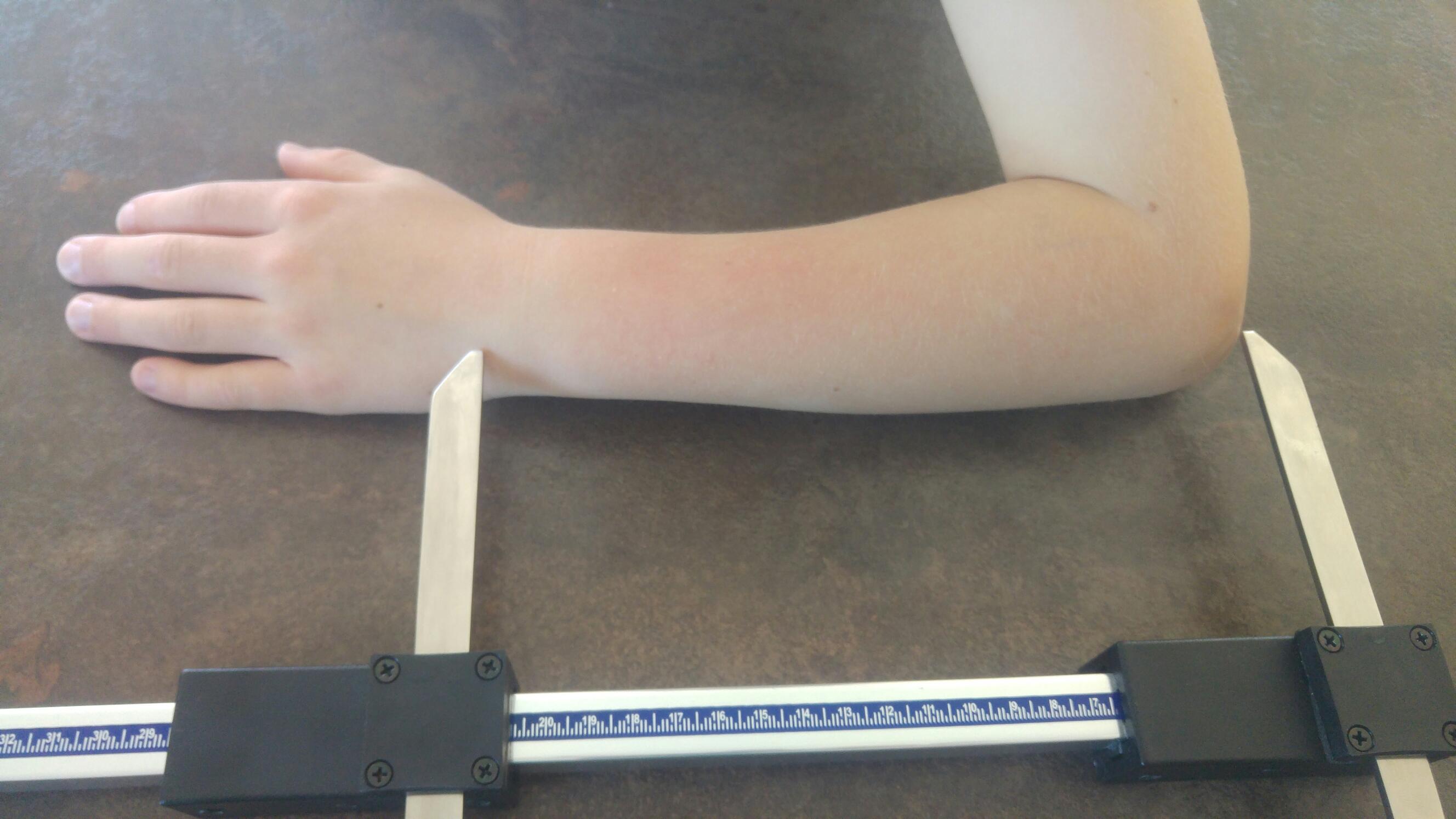
*

**Half of the arm span measurement / Tape measure**

**Measurement:**

- Position the child in a recumbent position.
- One operator places the extended upper limb in a 90° angle with the trunk; palms are positioned upwards, with extended fingers.
- The second operator places the tape measure from the finger ends towards the sternum.
- The second operator reads the measure in cm (+/- 0.5cm).

**Height extrapolation Formulas (Gauld et al.):**

- Boys:

**Height (cm)= 0.829 x (HoAS x 2) + 0.721 x age (in years) +16.258**

- Girls:

**Height (cm) = 0.619 x (HoAS x 2) + 1.593 x age (in years) +36.976**

*HoAS = Half of the arm span*


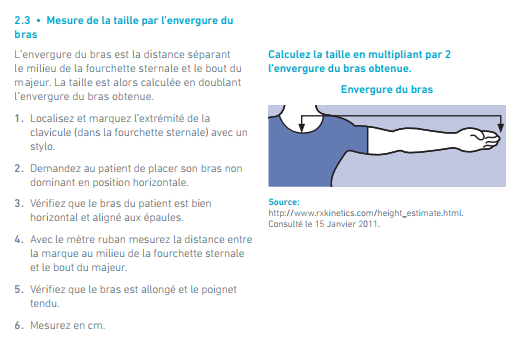


**Sum of body segment length measurements alongside the recumbent body (head + trunk + lower limbs) with a tape measure**

**Measurement:**

- Position the child in a recumbent position. Arms are alongside the body.
  - The line formed between the upper side of the two shoulders is perpendicular to the body axis.
  - The line formed between the two greater trochanters is perpendicular to the body axis.
  - The line formed between both heels is perpendicular to the body axis (feet are positioned with a 90° flexion of the ankle).
- One operator ensures that the child’s body remains in the same position.
- The second operator places the tape measure alongside the body, on the bed, on one side of the child, under any line connected to indwelling devices (e.g. central lines, drains, etc.) or cable connected to monitoring devices (e.g. heart rate, SpO2 monitors)
- The second operator performs 3 successive measurements of different body segments and reads the measure in cm (+/- 0.5cm):
  - Segment 1: head. From an imaginary line (perpendicular to the body axis) at the vertex level, towards the upper part of the shoulder.
  - Segment 2: trunk. From the upper part of the shoulder to the greater trochanter.
  - Segment 3: lower limb. From the greater trochanter to the heel.

**Height extrapolation Formulas:**

**Height (cm) = head (cm) + trunk (cm) + limb (cm)**


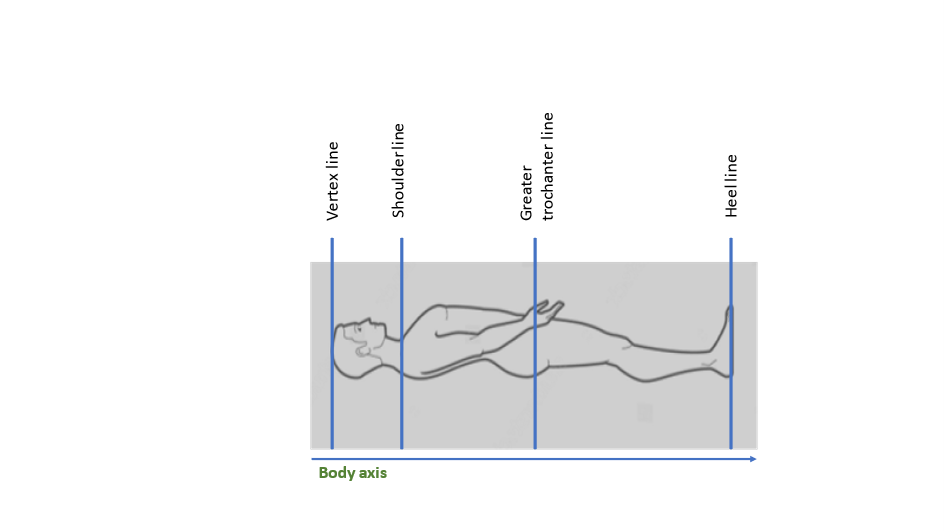


**Length measurement alongside the recumbent body with a tape measure**

**Measurement:**

- Position the child in a recumbent position. Arms are alongside the body. Feet are positioned with a 90° flexion of the ankle.
- One operator ensures that the child’s body remains in the same position.
- The second operator places the tape measure alongside the body, on the bed, on one side of the child, under any line connected to indwelling devices (e.g. central lines, drains, etc.) or cable connected to monitoring devices (e.g. heart rate, SpO2 monitors)
- Height is measured between a virtual line passing the head vertex and perpendicular to the body axis, and a virtual line passing the heels.
- The second operator performs the measurement and reads the measure in cm (+/- 0.5cm):


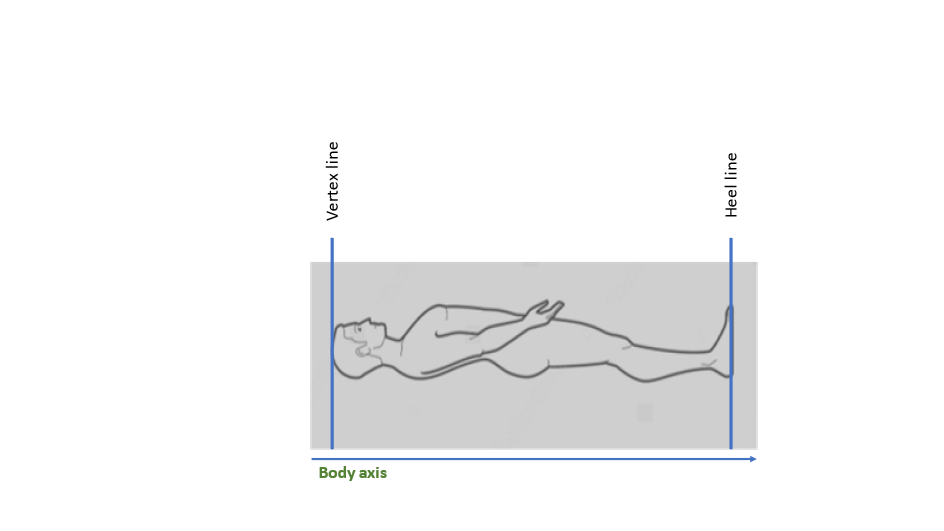


**Estimation from parents’ report**

Ask one or both parents if they know the length or height of their child. If their answers differ, calculate the mean of the two values.

**Last height found in the child’s health records / medical file**

Look for previous measured heights or length collected in the child’s heath records (or in his personal file if available)

**Estimation from a length board in children <2 years**

Use the same technique than the one recommended as per WHO gold standard, adapt to the clinical condition of the child and to the indwelling device.

**Extrapolation from previous measurements allowing for height growth chart projection.**

Select a growth chart adapted to the child’s gender and age group (WHO growth charts can be used or national ones)

Extrapolate actual height prolonging the height growth curve following the same z-score for age.


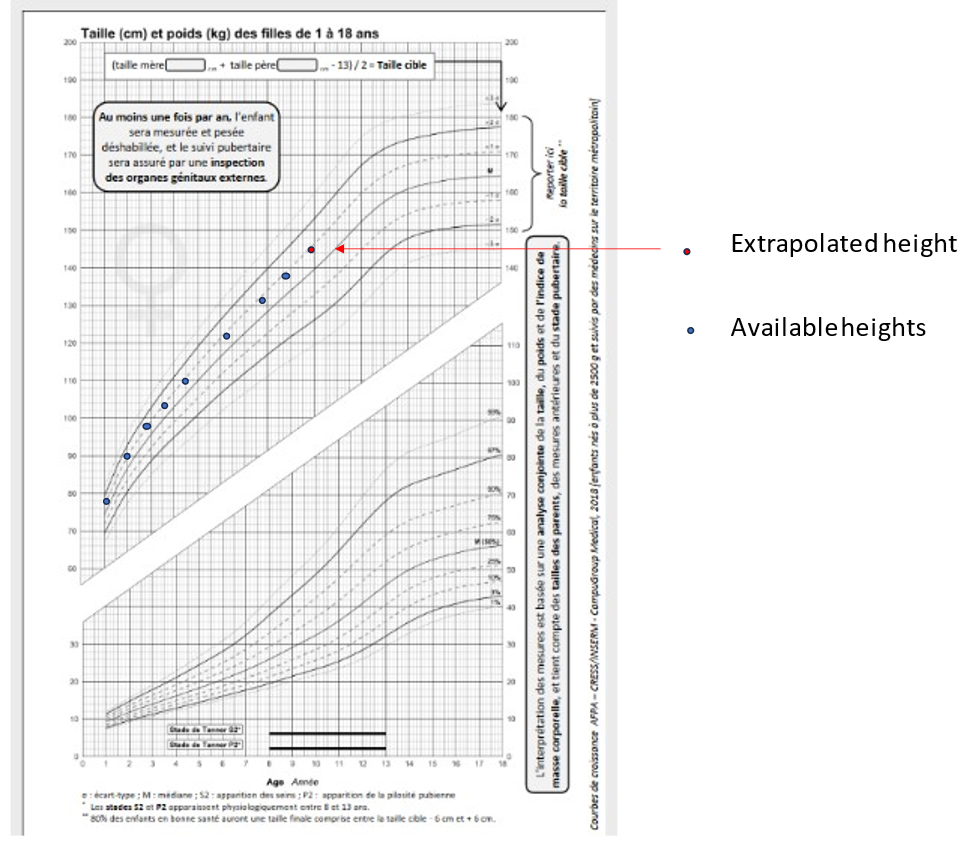


**Extrapolation from a hypothetical identical height for age z-score to the actual or most recent weight for age z-score**

Select a growth chart adapted to the child’s gender and age group (WHO growth charts can be used or national ones)

Identify the current weight for age z-score.

Plot the corresponding height for age z-score: z-score for age should be identical between weight and height.


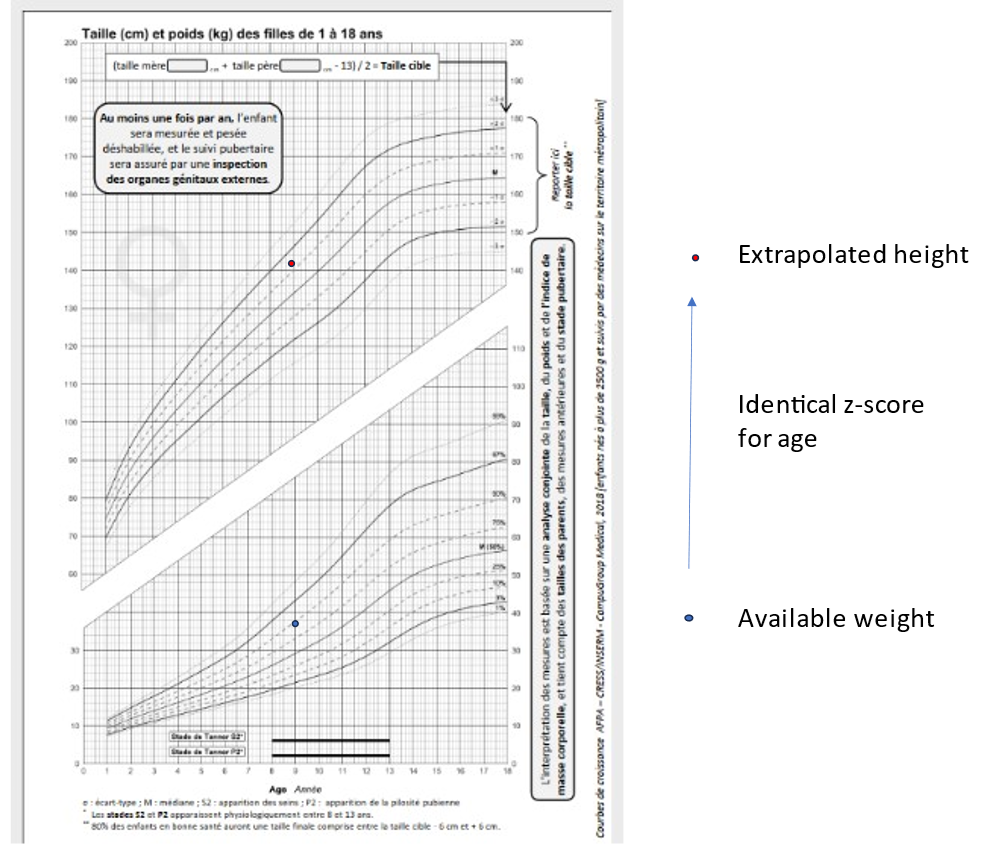


**Extrapolation from genetic parental height target**

- Both parents’ heights are collected.
- The target height of the child is calculated using the following formulas:
  - Boys: target height (in cm) = (mother’s height + father’s height + 13) /2
  - Girls: target height (in cm) = (mother’s height + father’s height - 13) /2
- The 18-year target height z-score is located on height growth curve (WHO curves or national ones)
- Similar height for age z-score is extrapolated.


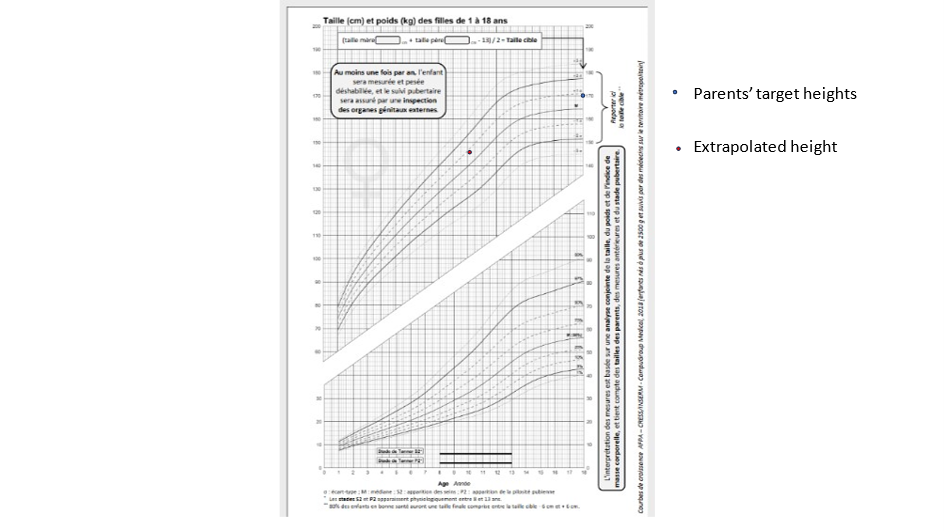


**Height measurement / WHO gold standard method**

**Children < 2 years**

**Length board**

- Position the child in a recumbent position.
- One operator holds the child’s head toward the upper part of the length board.
- The second operator maintains the child recumbent with his legs extended. Feet are positioned with a 90° angle of the ankle.
- The second operator positions the mobile part of the length board towards the heels and reads the measure (+/- 0.1cm).


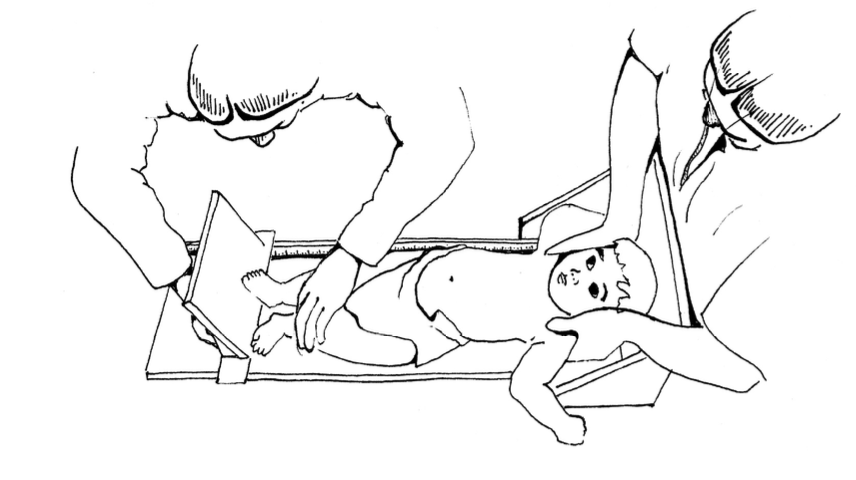


http://www.who.int/childgrowth/training/pratique_peser.pdf?ua=1

**Height measurement / WHO gold standard method**

**Children > 2 years**

**Stadiometer**

- A height board (also called “stadiometer”) is mounted at a right angle between a level floor and against a straight, vertical surface such as a wall or pillar.
- The child stands upright, one operator ensures that knees are extended.
- The second operator places the mobile part of the stadiometer on the vertex of the head and reads the measure (+/- 0.1cm)


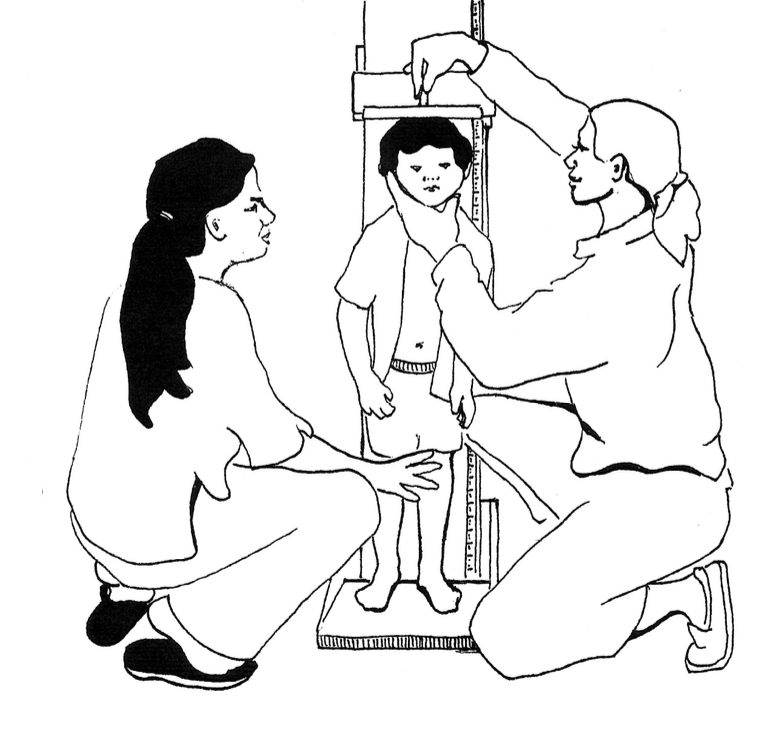


http://www.who.int/childgrowth/training/pratique_peser.p
