# Supplementary material for: Accurate height and length estimation in hospitalized children not fulfilling WHO criteria for standard measurement: a multicenter prospective study
Source: Eur J Pediatr. 2024 Jul 25;183(10):4275–86. doi: 10.1007/s00431-024-05692-3 (PMC11413069; doi:10.1007/s00431-024-05692-3)
Supplement: Supplementary file 3 — Supplementary file3 (DOCX 1326 KB) [file 431_2024_5692_MOESM3_ESM.docx]

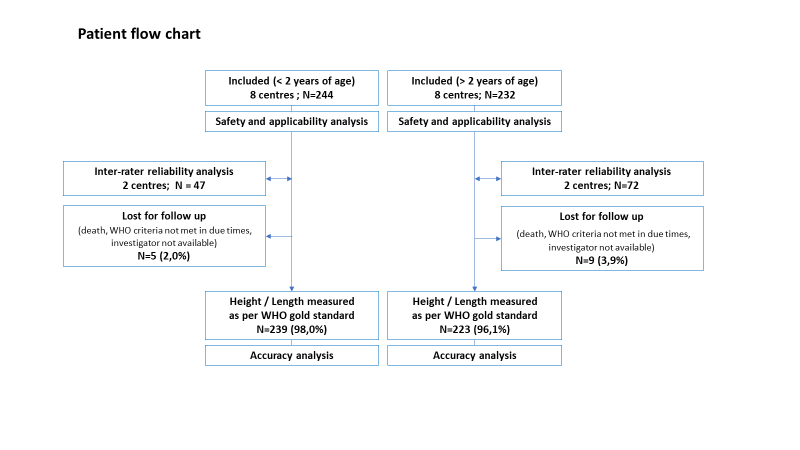


Supplemental figure 1: patient flow chart

Supplemental figure 2: Bland and Altman graphs, by method and length (surrogate of age) in children < 2 years


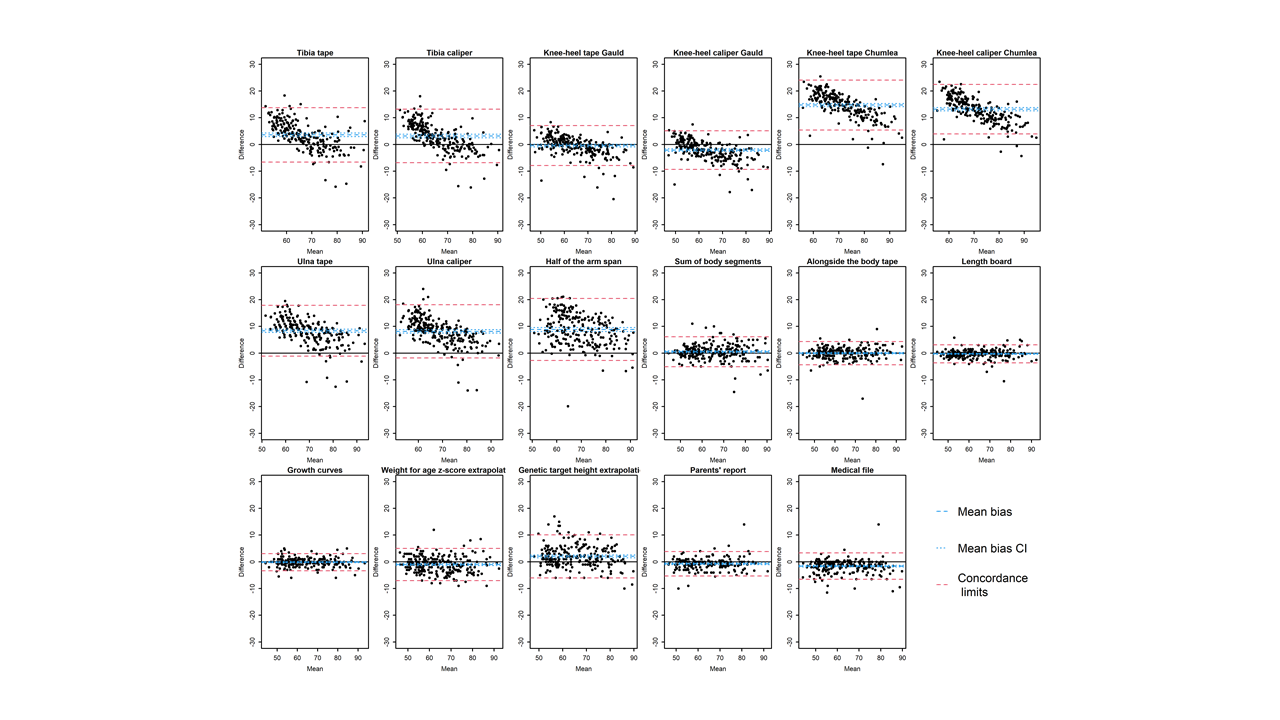


Supplemental figure 3: Bland et Altman graphs, by method and height (surrogate of age) in children ≥ 2 years
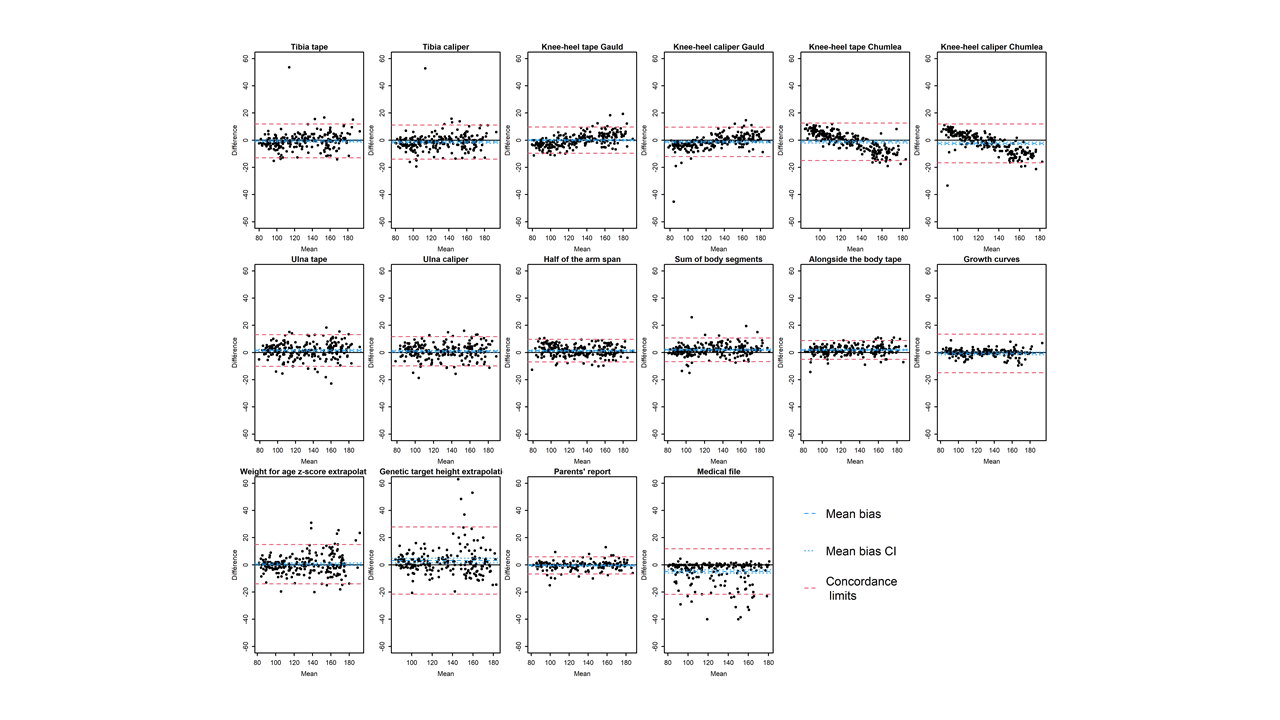


Supplemental table 3: Patients’ characteristics (population that was measured according to WHO gold standard)

| **Age group (years)** | **<2** | **≥2** | **Total** |
| --- | --- | --- | --- |
|  | N = 239 | N = 223 | N = 462 |
| Gender |  |  |  |
| Male | 125 (52.3%) | 130 (58.3%) | 255 (55.2%) |
| Female | 114 (47.7%) | 93 (41.7%) | 207 (44.8%) |
| Age (months) |  |  |  |
| Median | 5.00 | 99.00 | 20.00 |
| Q1-Q3 | 2.00 - 11.00 | 51.50 - 167.50 | 4.70 - 92.00 |
| Min-Max | 1.00 - 23.00 | 24.00 - 213.00 | 1.00 - 213.00 |
| Weight (kg) |  |  |  |
| Median | 6.21 | 26.00 | 11.00 |
| Q1-Q3 | 4.40 - 8.40 | 16.00 - 49.00 | 6.20 - 25.00 |
| N | 238 | 222 | 460 |

Supplemental table 4 Patients’ characteristics (overall population)

| **Age group (Years)** | **<2** | **≥2** | **Total** |
| --- | --- | --- | --- |
|  | N = 244 | N = 232 | N = 476 |
| PELOD2 score |  |  |  |
| Median | 5.00 | 4.00 | 4.50 |
| Q1-Q3 | 2.75 - 8.00 | 2.00 - 7.00 | 2.00 - 8.00 |
| N | 240 | 226 | 466 |
| Admission diagnosis |  |  |  |
| Respiratory | 109 (44.7%) | 47 (20.3%) | 156 (32.8%) |
| Hemodynamics | 6 ( 2.5%) | 12 ( 5.2%) | 18 ( 3.8%) |
| Infectious disease | 3 ( 1.2%) | 3 ( 1.3%) | 6 ( 1.3%) |
| Neurology | 15 ( 6.1%) | 25 (10.8%) | 40 ( 8.4%) |
| Métabolism/endocrinology/nephrology | 3 ( 1.2%) | 20 ( 8.6%) | 23 ( 4.8%) |
| Digestive | 2 ( 0.8%) | 7 ( 3.0%) | 9 ( 1.9%) |
| Orthopedics | 0 ( 0.0%) | 0 ( 0.0%) | 0 ( 0.0%) |
| Abdominopelvic | 24 ( 9.8%) | 40 (17.2%) | 64 (13.4%) |
| Neurosurgery | 7 ( 2.9%) | 19 ( 8.2%) | 26 ( 5.5%) |
| Head and neck | 9 ( 3.7%) | 5 ( 2.2%) | 14 ( 2.9%) |
| Cardiology | 65 (26.6%) | 49 (21.1%) | 114 (23.9%) |
| Other | 1 ( 0.4%) | 5 ( 2.2%) | 6 ( 1.3%) |
| Time between admission and inclusion (days) |  |  |  |
| Median | 2.00 | 1.00 | 1.00 |
| Q1-Q3 | 1.00 - 4.00 | 1.00 - 3.00 | 1.00 - 4.00 |
| Ventilation |  |  |  |
| Invasive | 113 (46.3%) | 84 (36.2%) | 197 (41.4%) |
| Non invasive | 102 (41.8%) | 51 (22.0%) | 153 (32.1%) |
| None | 29 (11.9%) | 97 (41.8%) | 126 (26.5%) |
| Invasive ventilation |  |  |  |
| Endotracheal tube | 112 (99.1%) | 81 (96.4%) | 193 (98.0%) |
| Tracheostomy | 1 ( 0.9%) | 3 ( 3.6%) | 4 ( 2.0%) |
| (col %) | N = 113 | N = 84 | N = 197 |
| Sedation |  |  |  |
| Yes | 114 (46.7%) | 92 (39.7%) | 206 (43.3%) |
| Position |  |  |  |
| Supine position | 237 (97.1%) | 226 (97.8%) | 463 (97.5%) |
| Prone position | 0 ( 0.0%) | 1 ( 0.4%) | 1 ( 0.2%) |
| Lateral decubitus | 7 ( 2.9%) | 4 ( 1.7%) | 11 ( 2.3%) |
| (col %) | N = 244 | N = 231 | N = 475 |
| Bed Angle / Head up |  |  |  |
| 0° | 16 ( 6.6%) | 16 ( 6.9%) | 32 ( 6.7%) |
| 0 à 30° | 211 (86.5%) | 146 (62.9%) | 357 (75.0%) |
| 30 à 45° | 16 ( 6.6%) | 58 (25.0%) | 74 (15.5%) |
| 45 à 90° | 1 ( 0.4%) | 12 ( 5.2%) | 13 ( 2.7%) |
| Intravascular Catheters (arterial / veinous) |  |  |  |
| Yes | 219 (89.8%) | 218 (94.4%) | 437 (92.0%) |
| (col %) | N = 244 | N = 231 | N = 475 |
| Peripheral scalp vein |  |  |  |
| Yes | 2 ( 0.9%) | 1 ( 0.5%) | 3 ( 0.7%) |
| (col %) | N = 219 | N = 217 | N = 436 |
| Peripheral other |  |  |  |
| Yes | 165 (75.3%) | 171 (78.8%) | 336 (77.1%) |
| (col %) | N = 219 | N = 217 | N = 436 |
| Jugular |  |  |  |
| Yes | 91 (41.6%) | 90 (41.5%) | 181 (41.5%) |
| (col %) | N = 219 | N = 217 | N = 436 |
| Sub-clavian |  |  |  |
| Yes | 45 (20.5%) | 52 (24.0%) | 97 (22.2%) |
| (col %) | N = 219 | N = 217 | N = 436 |
| Femoral |  |  |  |
| Yes | 71 (32.4%) | 48 (22.1%) | 119 (27.3%) |
| (col %) | N = 219 | N = 217 | N = 436 |
| Intra osseous |  |  |  |
| Yes | 0 ( 0.0%) | 1 ( 0.5%) | 1 ( 0.2%) |
| (col %) | N = 219 | N = 217 | N = 436 |
| Radial |  |  |  |
| Yes | 16 ( 7.3%) | 61 (28.1%) | 77 (17.7%) |
| (col %) | N = 219 | N = 217 | N = 436 |
| Arterial |  |  |  |
| Yes | 76 (35.0%) | 99 (45.6%) | 175 (40.3%) |
| (col %) | N = 217 | N = 217 | N = 434 |
| Veinous |  |  |  |
| Yes | 215 (99.1%) | 215 (99.1%) | 430 (99.1%) |
| (col %) | N = 217 | N = 217 | N = 434 |
| Urinary catheter |  |  |  |
| Yes | 144 (59.0%) | 171 (73.7%) | 315 (66.2%) |
| Drains |  |  |  |
| Yes | 89 (36.5%) | 110 (47.4%) | 199 (41.8%) |
| Thoracic |  |  |  |
| Yes | 70 (78.7%) | 63 (57.3%) | 133 (66.8%) |
| (col %) | N = 89 | N = 110 | N = 199 |
| Abdominal |  |  |  |
| Yes | 18 (20.2%) | 40 (36.4%) | 58 (29.1%) |
| (col %) | N = 89 | N = 110 | N = 199 |
| Head/Neck |  |  |  |
| Yes | 3 ( 3.4%) | 12 (10.9%) | 15 ( 7.5%) |
| (col %) | N = 89 | N = 110 | N = 199 |
| Limbs |  |  |  |
| Yes | 0 ( 0.0%) | 0 ( 0.0%) | 0 ( 0.0%) |
| (col %) | N = 89 | N = 110 | N = 199 |
| Other |  |  |  |
| Yes | 0 ( 0.0%) | 0 ( 0.0%) | 0 ( 0.0%) |
| (col %) | N = 88 | N = 110 | N = 198 |
| Gastric (jejunal) tube |  |  |  |
| Yes | 181 (74.2%) | 80 (34.5%) | 261 (54.8%) |
| Gastrostomy/Jejunostomy |  |  |  |
| Yes | 12 ( 4.9%) | 15 ( 6.5%) | 27 ( 5.7%) |
| Stoma |  |  |  |
| Yes | 4 ( 1.6%) | 2 ( 0.9%) | 6 ( 1.3%) |
| Intracranial pressure monitoring |  |  |  |
| Yes | 6 ( 2.5%) | 11 ( 4.8%) | 17 ( 3.6%) |
| (col %) | N = 244 | N = 231 | N = 475 |
| Regional anesthesia catheter |  |  |  |
| Yes | 8 ( 3.3%) | 14 ( 6.1%) | 22 ( 4.6%) |
| (col %) | N = 244 | N = 231 | N = 475 |
| Dressings: head |  |  |  |
| Yes | 10 ( 4.1%) | 17 ( 7.4%) | 27 ( 5.7%) |
| (col %) | N = 244 | N = 231 | N = 475 |
| Other dressings |  |  |  |
| Yes | 109 (44.7%) | 106 (45.9%) | 215 (45.3%) |
| (col %) | N = 244 | N = 231 | N = 475 |
| Neck |  |  |  |
| Yes | 16 (14.8%) | 11 (10.4%) | 27 (12.6%) |
| (col %) | N = 108 | N = 106 | N = 214 |
| Thorax |  |  |  |
| Yes | 68 (63.0%) | 64 (60.4%) | 132 (61.7%) |
| (col %) | N = 108 | N = 106 | N = 214 |
| Abdominal |  |  |  |
| Yes | 13 (12.0%) | 26 (24.5%) | 39 (18.2%) |
| (col %) | N = 108 | N = 106 | N = 214 |
| Limbs |  |  |  |
| Yes | 28 (25.7%) | 29 (27.4%) | 57 (26.5%) |
| (col %) | N = 109 | N = 106 | N = 215 |
| Other |  |  |  |
| Yes | 2 ( 1.9%) | 2 ( 1.9%) | 4 ( 1.9%) |
| (col %) | N = 108 | N = 106 | N = 214 |
| Other devices |  |  |  |
| Yes | 14 ( 5.7%) | 24 (10.3%) | 38 ( 8.0%) |
| Peritoneal dialysis |  |  |  |
| Yes | 10 (71.4%) | 8 (33.3%) | 18 (47.4%) |
| (col %) | N = 14 | N = 24 | N = 38 |
| Renal replacement therapy |  |  |  |
| Yes | 5 (35.7%) | 13 (54.2%) | 18 (47.4%) |
| (col %) | N = 14 | N = 24 | N = 38 |
| ECMO ECLS (extracorporeal life support) |  |  |  |
| Yes | 2 (14.3%) | 4 (16.7%) | 6 (15.8%) |
| (col %) | N = 14 | N = 24 | N = 38 |
| Other |  |  |  |
| Yes | 1 ( 7.1%) | 2 ( 8.3%) | 3 ( 7.9%) |
| (col %) | N = 14 | N = 24 | N = 38 |
| Other devices |  |  |  |
| Yes | 5 ( 2.0%) | 9 ( 3.9%) | 14 ( 2.9%) |
| Cast & splints |  |  |  |
| Yes | 0 ( 0.0%) | 1 (11.1%) | 1 ( 7.1%) |
| (col %) | N = 5 | N = 9 | N = 14 |
| Corset |  |  |  |
| Yes | 0 ( 0.0%) | 2 (22.2%) | 2 (14.3%) |
| (col %) | N = 5 | N = 9 | N = 14 |
| Traction |  |  |  |
| Yes | 0 ( 0.0%) | 0 ( 0.0%) | 0 ( 0.0%) |
| (col %) | N = 5 | N = 9 | N = 14 |
| Cervical collar |  |  |  |
| Yes | 0 ( 0.0%) | 0 ( 0.0%) | 0 ( 0.0%) |
| (col %) | N = 5 | N = 9 | N = 14 |

Supplemental table 5: body segment measurements results

| **Age group (years)** | **<2** | **≥2** |
| --- | --- | --- |
| TIBIA Tape measure (cm) |  |  |
| Median | 10.50 | 28.50 |
| Q1-Q3 | 9.00 - 12.00 | 20.50 - 35.00 |
| N | 239 | 223 |
| TIBIA caliper (cm) |  |  |
| Median | 10.00 | 28.00 |
| Q1-Q3 | 9.00 - 12.05 | 20.10 - 35.00 |
| N | 239 | 223 |
| Knee-Heel Tape measure (cm) |  |  |
| Median | 16.50 | 39.00 |
| Q1-Q3 | 14.50 - 19.50 | 30.00 - 49.00 |
| N | 239 | 223 |
| Knee-Heel caliper (cm) |  |  |
| Median | 15.50 | 39.00 |
| Q1-Q3 | 14.00 - 18.55 | 29.55 - 48.50 |
| N | 239 | 223 |
| ULNA Tape measure (cm) |  |  |
| Median | 9.00 | 19.50 |
| Q1-Q3 | 8.00 - 10.50 | 15.12 - 24.50 |
| N | 239 | 222 |
| ULNA Caliper (cm) |  |  |
| Median | 9.00 | 19.40 |
| Q1-Q3 | 8.10 - 10.30 | 15.22 - 24.45 |
| N | 238 | 222 |
| Half of the arm span Tape measure (cm) |  |  |
| Median | 30.50 | 65.00 |
| Q1-Q3 | 27.50 - 36.00 | 51.00 - 79.50 |
| N | 237 | 220 |
| Body segment Head (cm) |  |  |
| Median | 14.50 | 21.50 |
| Q1-Q3 | 13.00 - 16.50 | 19.50 - 23.50 |
| N | 239 | 223 |
| Body segment Trunk (cm) |  |  |
| Median | 24.50 | 44.00 |
| Q1-Q3 | 21.50 - 27.00 | 35.00 - 52.00 |
| N | 239 | 223 |
| Body segment lower limb (cm) |  |  |
| Median | 25.00 | 66.30 |
| Q1-Q3 | 21.00 - 29.25 | 49.50 - 83.00 |
| N | 239 | 223 |
| Number of available plots on growth charts |  |  |
| Median | 5.00 | 8.00 |
| Q1-Q3 | 3.00 - 8.00 | 5.00 - 12.00 |
| N | 225 | 211 |
| Mother’s height (cm) |  |  |
| Median | 165.00 | 163.00 |
| Q1-Q3 | 160.00 - 170.00 | 160.00 - 168.00 |
| N | 229 | 210 |
| Father’s height (cm) |  |  |
| Median | 178.00 | 177.00 |
| Q1-Q3 | 173.00 - 182.00 | 172.00 - 182.00 |
| N | 229 | 206 |
| Genetic target height (parental) (cm) |  |  |
| Median | 173.00 | 172.50 |
| Q1-Q3 | 165.50 - 179.00 | 163.62 - 178.50 |
| N | 229 | 206 |

Supplemental figure 4: Boxplot of anthropometric measurements, by method and age group


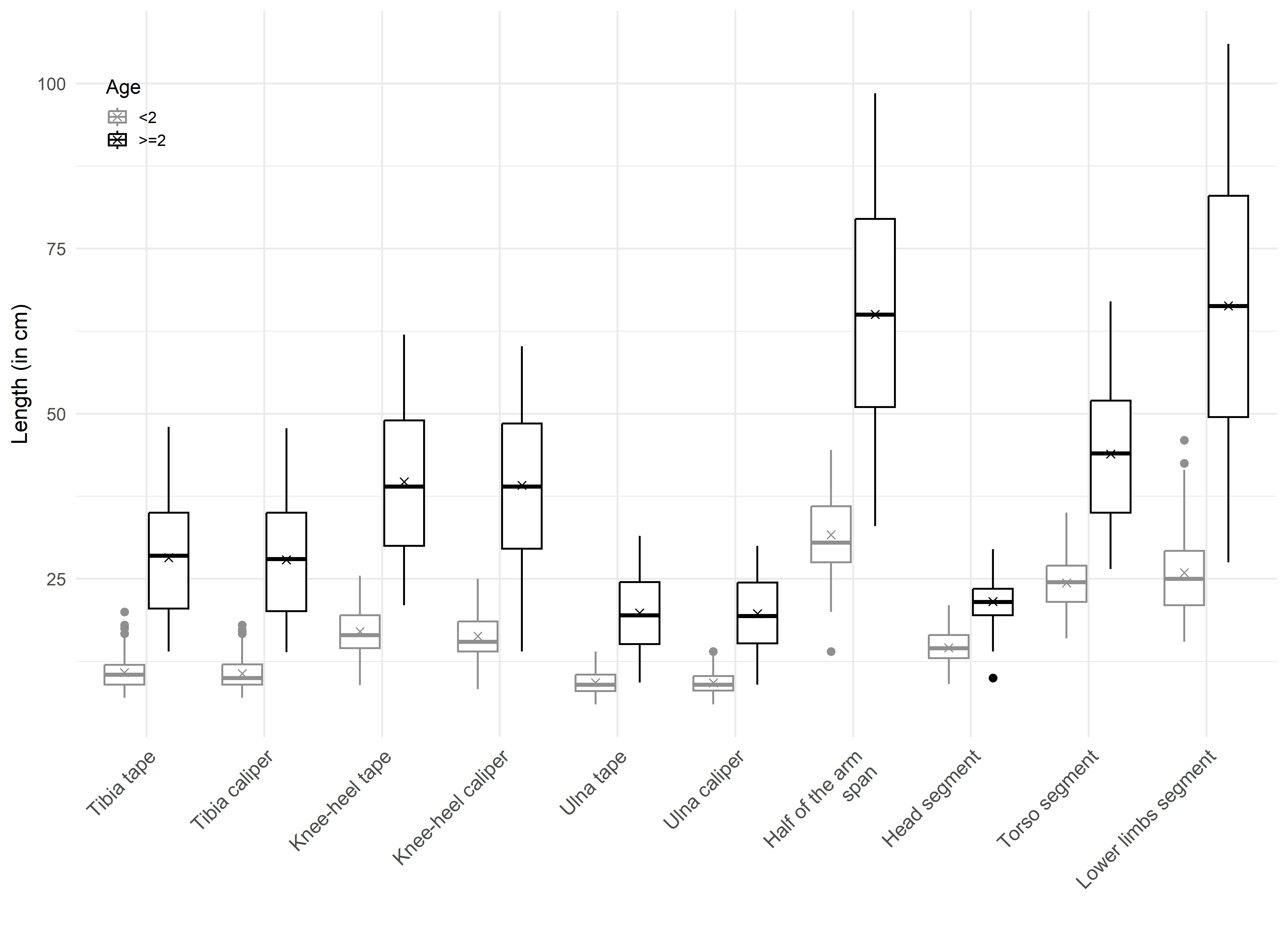


**Supplemental Table 6: Height or Length estimation or extrapolation results**

| **Age group (years)** | **<2** | **≥2** |
| --- | --- | --- |
| Height / Length (in cm) |  |  |
| TIBIA Tape measure (cm) |  |  |
| Median | 66.54 | 129.33 |
| Q1-Q3 | 62.81 - 72.48 | 100.13 - 155.93 |
| N | 239 | 223 |
| TIBIA caliper (cm) |  |  |
| Median | 65.58 | 126.80 |
| Q1-Q3 | 62.08 - 72.54 | 99.25 - 155.61 |
| N | 239 | 223 |
| Knee-Heel Tape measure Gauld (1) |  |  |
| Median | 62.35 | 128.14 |
| Q1-Q3 | 57.15 - 70.17 | 99.27 - 158.95 |
| N | 239 | 223 |
| Knee-Heel caliper Gauld (cm) |  |  |
| Median | 60.32 | 127.29 |
| Q1-Q3 | 55.95 - 68.06 | 98.19 - 157.64 |
| N | 239 | 223 |
| Knee-Heel tape measure Chumlea (cm) |  |  |
| Median | 78.28 | 127.12 |
| Q1-Q3 | 73.31 - 84.06 | 107.14 - 148.56 |
| N | 239 | 223 |
| Knee-Heel caliper Chumlea (cm) |  |  |
| Median | 76.53 | 127.12 |
| Q1-Q3 | 72.06 - 82.56 | 106.14 - 147.51 |
| N | 239 | 223 |
| ULNA tape measure (cm) |  |  |
| Median | 70.65 | 128.81 |
| Q1-Q3 | 67.27 - 77.66 | 103.74 - 160.37 |
| N | 239 | 222 |
| ULNA Caliper (cm) |  |  |
| Median | 70.82 | 128.86 |
| Q1-Q3 | 66.63 - 77.30 | 103.89 - 159.96 |
| N | 238 | 222 |
| Half of the arm span (cm) |  |  |
| Median | 72.17 | 129.54 |
| Q1-Q3 | 66.90 - 79.15 | 105.42 - 158.50 |
| N | 237 | 220 |
| Sum of body segments |  |  |
| Median | 63.50 | 131.50 |
| Q1-Q3 | 56.00 - 73.00 | 103.25 - 160.50 |
| N | 239 | 223 |
| Alongside the body tape measure (cm) |  |  |
| Median | 63.00 | 131.00 |
| Q1-Q3 | 55.50 - 72.50 | 104.10 - 160.75 |
| N | 233 | 211 |
| Infantometer (Length board) (cm) |  |  |
| Median | 61.75 |  |
| Q1-Q3 | 55.00 - 72.00 |  |
| N | 232 |  |
| Growth charts (cm) |  |  |
| Median | 63.00 | 128.00 |
| Q1-Q3 | 55.00 - 72.00 | 103.75 - 156.00 |
| N | 225 | 212 |
| Weight for age z-score extrapolation (cm) |  |  |
| Mean (SD) | 63.29 ( 10.29) | 130.27 ( 30.20) |
| Median | 61.25 | 130.00 |
| Q1-Q3 | 55.00 - 70.00 | 103.50 - 158.00 |
| Min-Max | 46.00 - 91.00 | 80.00 - 203.00 |
| N | 238 | 223 |
| Genetic target Height extrapolation (cm) |  |  |
| Median | 65.00 | 134.00 |
| Q1-Q3 | 57.00 - 74.00 | 104.25 - 161.00 |
| N | 229 | 206 |
| Parents’ report (cm) |  |  |
| Median | 62.00 | 133.50 |
| Q1-Q3 | 55.00 - 71.50 | 105.25 - 160.00 |
| N | 189 | 182 |
| Medical chart (cm) |  |  |
| Median | 61.00 | 124.00 |
| Q1-Q3 | 54.00 - 71.00 | 98.50 - 150.50 |
| N | 229 | 213 |

**Supplemental table 7: WHO gold standard height or length measurement**

| **Age group (years)** | **<2** | **≥2** | **Total** |
| --- | --- | --- | --- |
|  | N = 239 | N = 223 | N = 462 |
| Height/Length : WHO gold standard (cm) |  |  |  |
| Median | 62.50 | 128.40 | 83.00 |
| Q1-Q3 | 55.00 - 72.25 | 104.00 - 157.20 | 62.00 - 127.50 |
| N | 239 | 223 | 462 |

Supplemental figure 5: Boxplot of calculated heights, by method and age group


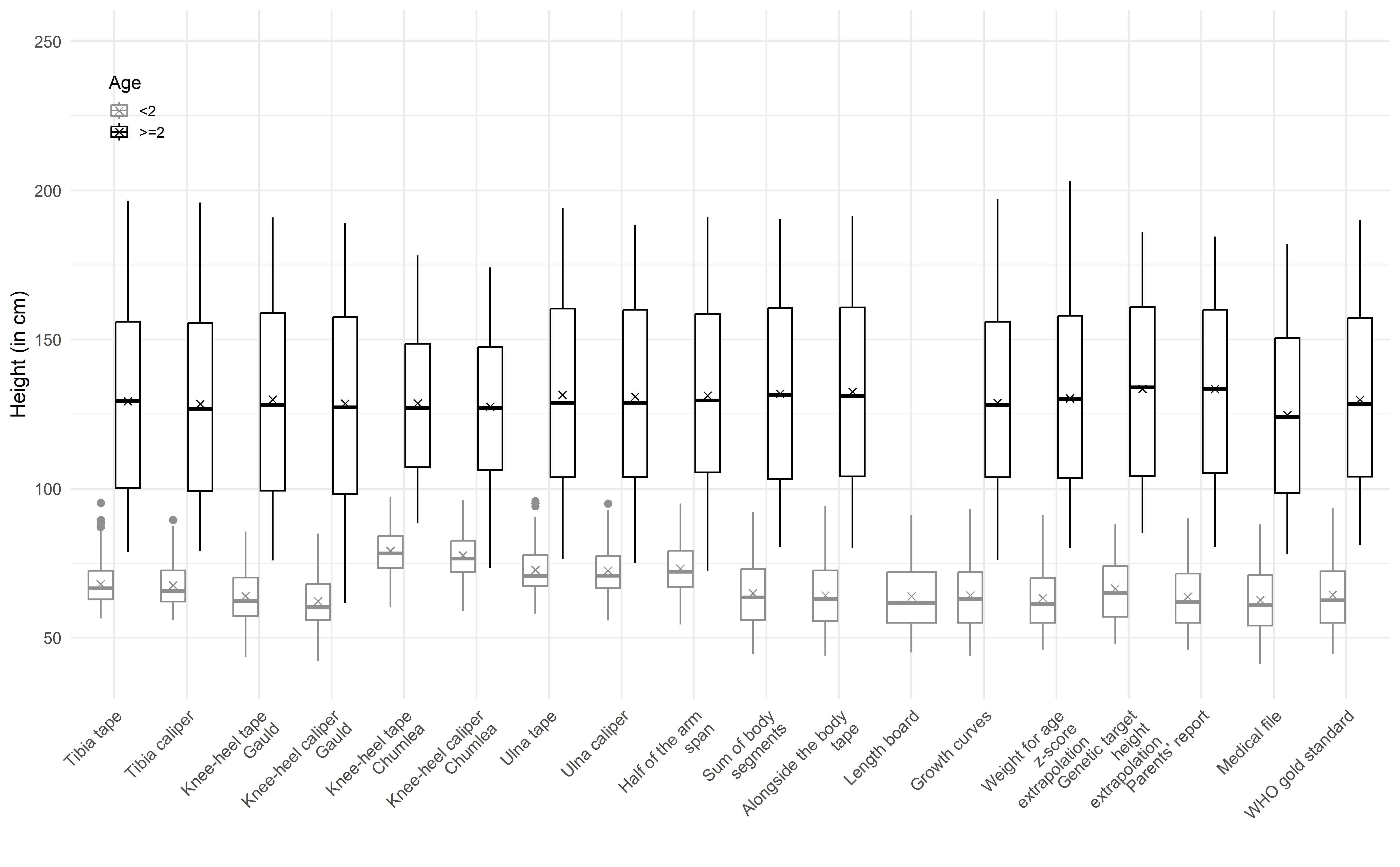


Supplemental Table 8: relative error according to extrapolation methods and age range

| **Age group (years)** | **<2** | **≥2** |
| --- | --- | --- |
| Relative Error (in %) |  |  |
| TIBIA Tape measure |  |  |
| Mean (SD) | 6.82 ( 8.98) | -0.51 ( 5.78) |
| Median | 6.02 | -0.55 |
| Q1-Q3 | 0.59 - 13.53 | -3.30 - 1.64 |
| Min-Max | -18.10 - 36.70 | -14.58 - 61.68 |
| N | 239 | 223 |
| TIBIA caliper |  |  |
| Mean (SD) | 6.14 ( 8.80) | -1.19 ( 5.84) |
| Median | 5.34 | -1.33 |
| Q1-Q3 | -0.11 - 12.32 | -3.89 - 0.93 |
| Min-Max | -18.97 - 36.15 | -17.12 - 60.73 |
| N | 239 | 223 |
| Knee Heel tape measure Gauld |  |  |
| Mean (SD) | 0.00 ( 5.79) | -0.48 ( 3.88) |
| Median | -0.23 | -0.44 |
| Q1-Q3 | -3.19 - 3.45 | -3.16 - 2.11 |
| Min-Max | -23.70 - 16.69 | -12.81 - 11.74 |
| N | 239 | 223 |
| Knee heel caliper Gauld |  |  |
| Mean (SD) | -2.72 ( 5.47) | -1.53 ( 4.73) |
| Median | -2.98 | -1.21 |
| Q1-Q3 | -6.04 - 0.70 | -3.85 - 1.08 |
| Min-Max | -26.25 - 14.07 | -42.35 - 9.37 |
| N | 239 | 223 |
| Knee Heel tape measure Chumlea |  |  |
| Mean (SD) | 24.52 (10.71) | 0.16 ( 5.56) |
| Median | 24.36 | -0.30 |
| Q1-Q3 | 16.77 - 32.24 | -4.36 - 4.17 |
| Min-Max | -8.12 - 52.66 | -10.87 - 13.96 |
| N | 239 | 223 |
| Knee heel caliper Chumlea |  |  |
| Mean (SD) | 22.08 (10.46) | -0.79 ( 5.76) |
| Median | 21.38 | -1.20 |
| Q1-Q3 | 13.70 - 29.48 | -5.21 - 3.56 |
| Min-Max | -4.71 - 52.66 | -31.29 - 13.69 |
| N | 239 | 223 |
| ULNA tape measure |  |  |
| Mean (SD) | 14.27 ( 9.11) | 1.28 ( 4.64) |
| Median | 14.56 | 1.31 |
| Q1-Q3 | 8.72 - 19.36 | -1.26 - 3.99 |
| Min-Max | -14.54 - 38.99 | -13.62 - 14.39 |
| N | 239 | 222 |
| ULNA caliper |  |  |
| Mean (SD) | 14.00 ( 9.63) | 0.81 ( 4.38) |
| Median | 13.75 | 0.88 |
| Q1-Q3 | 7.50 - 19.12 | -1.33 - 3.24 |
| Min-Max | -15.97 - 48.02 | -16.47 - 11.72 |
| N | 238 | 222 |
| Half of the arm span |  |  |
| Mean (SD) | 14.95 (10.98) | 1.24 ( 3.71) |
| Median | 13.88 | 0.98 |
| Q1-Q3 | 6.55 - 21.57 | -0.94 - 3.45 |
| Min-Max | -26.69 - 45.04 | -14.81 - 12.44 |
| N | 237 | 220 |
| Sum of body segments |  |  |
| Mean (SD) | 0.93 ( 4.35) | 1.54 ( 3.72) |
| Median | 0.82 | 1.40 |
| Q1-Q3 | -1.45 - 3.33 | -0.30 - 3.05 |
| Min-Max | -17.68 - 22.00 | -13.51 - 27.96 |
| N | 239 | 223 |
| Alongside the body tape measure |  |  |
| Mean (SD) | -0.02 ( 3.35) | 1.44 ( 2.83) |
| Median | 0.00 | 1.55 |
| Q1-Q3 | -1.77 - 1.82 | 0.00 - 3.21 |
| Min-Max | -20.73 - 11.84 | -15.16 - 7.63 |
| N | 233 | 211 |
| Length board (infantometer) |  |  |
| Mean (SD) | -0.41 ( 2.56) |  |
| Median | 0.00 |  |
| Q1-Q3 | -1.66 - 0.86 |  |
| Min-Max | -12.80 - 11.60 |  |
| N | 232 |  |
| Growth charts |  |  |
| Mean (SD) | -0.18 ( 2.64) | -0.39 ( 4.36) |
| Median | 0.00 | 0.00 |
| Q1-Q3 | -1.36 - 0.87 | -0.81 - 0.78 |
| Min-Max | -10.28 - 9.80 | -56.57 - 10.34 |
| N | 225 | 212 |
| Extrapolation of weight for age z-score |  |  |
| Mean (SD) | -1.39 ( 4.74) | 0.36 ( 5.41) |
| Median | -1.64 | 0.00 |
| Q1-Q3 | -4.74 - 1.57 | -2.81 - 2.86 |
| Min-Max | -12.31 - 21.43 | -16.88 - 25.20 |
| N | 238 | 223 |
| Genetic target extrapolation |  |  |
| Mean (SD) | 3.58 ( 7.04) | 2.92 (11.13) |
| Median | 2.13 | 0.93 |
| Q1-Q3 | -0.83 - 6.98 | -1.96 - 4.48 |
| Min-Max | -10.99 - 35.42 | -18.55 - 96.13 |
| N | 229 | 206 |
| Parents’ report |  |  |
| Mean (SD) | -1.12 ( 3.59) | -0.36 ( 2.57) |
| Median | -0.73 | 0.00 |
| Q1-Q3 | -2.78 - 0.00 | -1.19 - 0.67 |
| Min-Max | -17.86 - 18.92 | -14.02 - 9.45 |
| N | 189 | 182 |
| Medical file |  |  |
| Mean (SD) | -2.51 ( 3.87) | -3.60 ( 6.00) |
| Median | -1.86 | -0.71 |
| Q1-Q3 | -3.92 - 0.00 | -4.81 - 0.00 |
| Min-Max | -18.85 - 19.44 | -28.78 - 5.00 |
| N | 229 | 213 |

Supplemental table 9: Absolute mean relative error according to the age-group, tested against the reference value of 3.5M%

| **Method** | **Absolute mean relative error (%)** | **Upper 95% CI** | **p-value** |
| --- | --- | --- | --- |
| TIBIA tape measure |  |  |  |
| <2 years | 6.82 | ]. ; 7.77] | 1.000 |
| ≥2 years | 0.51 | ]. ; 1.12] | <0.001 |
| TIBIA Caliper |  |  |  |
| <2 years | 6.14 | ]. ; 7.07] | 1.000 |
| ≥2 years | 1.19 | ]. ; 1.8] | <0.001 |
| Knee-Heel tape measure Gauld |  |  |  |
| <2 years | 0 | ]. ; 0.62] | <0.001 |
| ≥2 years | 0.48 | ]. ; 0.88] | <0.001 |
| Knee-Heel caliper Gauld |  |  |  |
| <2 years | 2.72 | ]. ; 3.3] | 0.013 |
| ≥2 years | 1.53 | ]. ; 2.03] | <0.001 |
| Knee-Heel tape meas. Chumlea |  |  |  |
| <2 years | 24.52 | ]. ; 25.66] | 1.000 |
| ≥2 years | 0.16 | ]. ; 0.74] | <0.001 |
| Knee-Heel tape cal. Chumlea |  |  |  |
| <2 years | 22.08 | ]. ; 23.2] | 1.000 |
| ≥2 years | 0.79 | ]. ; 1.39] | <0.001 |
| ULNA tape measure |  |  |  |
| <2 years | 14.27 | ]. ; 15.24] | 1.000 |
| ≥2 years | 1.28 | ]. ; 1.76] | <0.001 |
| ULNA caliper |  |  |  |
| <2 years | 14 | ]. ; 15.03] | 1.000 |
| ≥2 years | 0.81 | ]. ; 1.27] | <0.001 |
| Half of the arm span |  |  |  |
| <2 years | 14.95 | ]. ; 16.13] | 1.000 |
| ≥2 years | 1.24 | ]. ; 1.63] | <0.001 |
| Sum of body segments |  |  |  |
| <2 years | 0.93 | ]. ; 1.4] | <0.001 |
| ≥2 years | 1.54 | ]. ; 1.93] | <0.001 |
| Alongside the body tape measure |  |  |  |
| <2 years | 0.02 | ]. ; 0.38] | <0.001 |
| ≥2 years | 1.44 | ]. ; 1.74] | <0.001 |
| Length board |  |  |  |
| <2 years | 0.41 | ]. ; 0.69] | <0.001 |
| ≥2 years | - | - | - |
| Growth chart |  |  |  |
| <2 years | 0.18 | ]. ; 0.47] | <0.001 |
| ≥2 years | 0.39 | ]. ; 0.86] | <0.001 |
| Weight for age z-score extrapol. |  |  |  |
| <2 years | 1.39 | ]. ; 1.9] | <0.001 |
| ≥2 years | 0.36 | ]. ; 0.93] | <0.001 |
| Genetic target height |  |  |  |
| <2 years | 3.58 | ]. ; 4.34] | 0.567 |
| ≥2 years | 2.92 | ]. ; 4.12] | 0.214 |
| Parent’s report |  |  |  |
| <2 years | 1.12 | ]. ; 1.55] | <0.001 |
| ≥2 years | 0.36 | ]. ; 0.66] | <0.001 |
| Medical file |  |  |  |
| <2 years | 2.51 | ]. ; 2.93] | <0.001 |
| ≥2 years | 3.6 | ]. ; 4.25] | 0.599 |

Supplemental figure 6: Height relative errors, by method and age ≥ 2 years (without outliers exclusion)


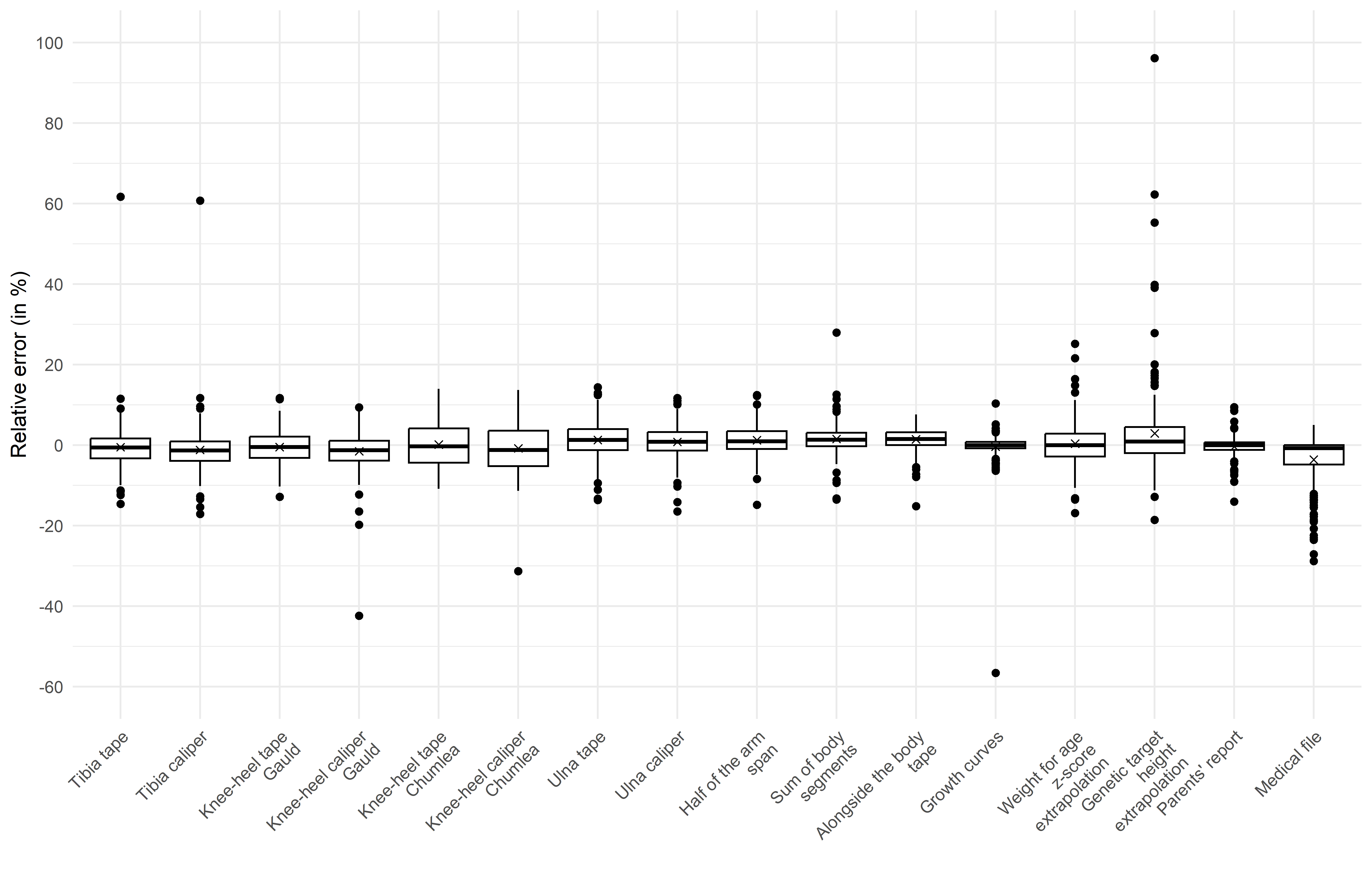


Supplemental table 10: Mean bias and concordance limits, age group below 2 years of age

| **Methods** | **Mean bias** | **95% CI** | **Concordance limits** |
| --- | --- | --- | --- |
| TIBIA tape measure | 3.61 | [2.95 ; 4.27] | [-6.57 ; 13.78] |
| TIBIA caliper | 3.17 | [2.52 ; 3.82] | [-6.84 ; 13.18] |
| Knee-Heel tape measure Gauld | -0.4 | [-0.88 ; 0.08] | [-7.86 ; 7.07] |
| Knee-Heel caliper Gauld | -2.11 | [-2.58 ; -1.64] | [-9.35 ; 5.13] |
| Knee-Heel tape meas. Chumlea | 14.74 | [14.13 ; 15.34] | [5.38 ; 24.1] |
| Knee-Heel caliper Chumlea | 13.21 | [12.61 ; 13.8] | [3.96 ; 22.45] |
| ULNA tape measure | 8.38 | [7.76 ; 8.99] | [-1.14 ; 17.89] |
| ULNA caliper | 8.15 | [7.51 ; 8.8] | [-1.79 ; 18.09] |
| Half of the arm span | 8.85 | [8.09 ; 9.6] | [-2.77 ; 20.46] |
| Sum of body segments | 0.53 | [0.16 ; 0.89] | [-5.09 ; 6.15] |
| Alongside the body tape measure | -0.02 | [-0.31 ; 0.26] | [-4.38 ; 4.33] |
| Length board | -0.26 | [-0.48 ; -0.04] | [-3.63 ; 3.1] |
| Growth chart | -0.14 | [-0.36 ; 0.07] | [-3.36 ; 3.08] |
| Weight for age z-score extrapol. | -1.01 | [-1.4 ; -0.61] | [-7.03 ; 5.02] |
| Genetic target extrapolation | 2.03 | [1.49 ; 2.56] | [-6.04 ; 10.09] |
| Parents’ report | -0.72 | [-1.05 ; -0.38] | [-5.29 ; 3.86] |
| Medical file | -1.61 | [-1.94 ; -1.28] | [-6.55 ; 3.33] |

Table 16 : Mean bias and concordance limits, age group above 2 years of age

| **Methods** | **Mean bias** | **95% CI** | **Concordance limit** |
| --- | --- | --- | --- |
| TIBIA tape measure | -0.53 | [-1.36 ; 0.3] | [-12.93 ; 11.87] |
| TIBIA caliper | -1.42 | [-2.25 ; -0.58] | [-13.93 ; 11.09] |
| Knee-Heel tape measure Gauld | 0.01 | [-0.63 ; 0.66] | [-9.64 ; 9.66] |
| Knee-Heel caliper Gauld | -1.28 | [-2 ; -0.55] | [-12.09 ; 9.53] |
| Knee-Heel tape meas. Chumlea | -1.19 | [-2.11 ; -0.27] | [-14.98 ; 12.6] |
| Knee-Heel caliper Chumlea | -2.35 | [-3.3 ; -1.39] | [-16.6 ; 11.91] |
| ULNA tape measure | 1.49 | [0.71 ; 2.28] | [-10.2 ; 13.18] |
| ULNA caliper | 0.94 | [0.22 ; 1.66] | [-9.83 ; 11.72] |
| Half of the arm span | 1.34 | [0.78 ; 1.9] | [-6.97 ; 9.66] |
| Sum of body segments | 1.99 | [1.41 ; 2.57] | [-6.7 ; 10.68] |
| Alongside the body tape measure | 1.9 | [1.42 ; 2.38] | [-5.03 ; 8.83] |
| Length board | - | - | - |
| Growth chart | -0.69 | [-1.67 ; 0.28] | [-14.89 ; 13.51] |
| Weight for age z-score extrapol. | 0.47 | [-0.5 ; 1.44] | [-14 ; 14.93] |
| Genetic target extrapolation | 3.17 | [1.45 ; 4.89] | [-21.51 ; 27.85] |
| Parents’ report | -0.42 | [-0.89 ; 0.05] | [-6.72 ; 5.88] |
| Medical file | -4.89 | [-6.03 ; -3.74] | [-21.62 ; 11.85] |

**Supplemental table 11: Relative errors and concordance correlation coefficient compared to WHO standard (CCC)- Age subgroup < 2 years**

| **Methods** | **% of absolute relative error <2%** | **% of absolute relative error <3.5%** | **CCC** | **95% CI** |
| --- | --- | --- | --- | --- |
| TIBIA tape measure | 15.06 | 29.71 | 0.78 | [0.74 ; 0.81] |
| TIBIA caliper | 18.83 | 32.64 | 0.79 | [0.75 ; 0.82] |
| Knee-Heel tape measure Gauld | 29.71 | 51.88 | 0.92 | [0.9 ; 0.94] |
| Knee-Heel caliper Gauld | 21.76 | 39.75 | 0.91 | [0.88 ; 0.92] |
| Knee-Heel tape meas. Chumlea | 0.84 | 2.09 | 0.37 | [0.32 ; 0.41] |
| Knee-Heel caliper Chumlea | 0.42 | 2.09 | 0.42 | [0.37 ; 0.46] |
| ULNA tape measure | 5.02 | 8.37 | 0.62 | [0.56 ; 0.66] |
| ULNA caliper | 4.2 | 8.82 | 0.61 | [0.56 ; 0.66] |
| Half of the arm span | 6.33 | 9.7 | 0.58 | [0.52 ; 0.63] |
| Sum of body segments | 42.68 | 64.85 | 0.96 | [0.95 ; 0.97] |
| Alongside the body tape meas. | 56.22 | 77.68 | 0.98 | [0.97 ; 0.98] |
| Length board | 71.98 | 86.21 | 0.99 | [0.98 ; 0.99] |
| Growth chart | 71.56 | 84.44 | 0.99 | [0.98 ; 0.99] |
| Weight for age z-sc. extrapol. | 36.97 | 51.26 | 0.95 | [0.94 ; 0.96] |
| Genetic target extrapolation | 34.06 | 49.34 | 0.9 | [0.88 ; 0.92] |
| Parents’ report | 55.56 | 72.49 | 0.97 | [0.96 ; 0.98] |
| Medical file | 50.66 | 69.43 | 0.96 | [0.95 ; 0.97] |

**Supplemental table 12: Relative errors and concordance correlation coefficient (CCC) compared to WHO standard - Age subgroup > 2 years**

| **Methods** | **% of absolute relative error <2%** | **% of relative error <3.5%** | **CCC** | **95% CI** |
| --- | --- | --- | --- | --- |
| TIBIA tape measure | 41.7 | 64.57 | 0.98 | [0.97 ; 0.98] |
| TIBIA caliper | 41.7 | 65.02 | 0.98 | [0.97 ; 0.98] |
| Knee-Heel tape measure Gauld | 39.46 | 66.82 | 0.99 | [0.98 ; 0.99] |
| Knee-Heel caliper Gauld | 43.05 | 64.13 | 0.98 | [0.98 ; 0.99] |
| Knee-Heel tape meas. Chumlea | 24.22 | 40.81 | 0.96 | [0.96 ; 0.97] |
| Knee-Heel caliper Chumlea | 20.18 | 39.91 | 0.96 | [0.95 ; 0.97] |
| ULNA tape measure | 35.59 | 59.46 | 0.98 | [0.97 ; 0.98] |
| ULNA caliper | 40.54 | 61.71 | 0.98 | [0.98 ; 0.99] |
| Half of the arm span | 45 | 67.27 | 0.99 | [0.98 ; 0.99] |
| Sum of body segments | 50.67 | 73.09 | 0.99 | [0.98 ; 0.99] |
| Alongside the body tape meas. | 51.18 | 75.83 | 0.99 | [0.99 ; 0.99] |
| Growth chart | 77.83 | 90.57 | 0.97 | [0.96 ; 0.98] |
| Weight for age z-sc. extrapol. | 36.77 | 58.3 | 0.97 | [0.96 ; 0.98] |
| Genetic target extrapolation | 33.01 | 53.88 | 0.9 | [0.88 ; 0.93] |
| Parents’ report | 74.73 | 89.01 | 0.99 | [0.99 ; 1] |
| Medical file | 60.56 | 69.48 | 0.94 | [0.93 ; 0.96] |

Supplemental table 13: Difficulty assessment of each method. Number of patients for whom difficulties were encountered during measurements

| **Age group (years)** | **<2** | **≥2** |
| --- | --- | --- |
| Difficulty assessment |  |  |
| TIBIA tape measure |  |  |
| Difficulties | 131 (53.7%) | 37 (15.9%) |
| Impossible | 0 ( 0.0%) | 0 ( 0.0%) |
| No difficulties | 113 (46.3%) | 195 (84.1%) |
| Missing data | 0 ( 0.0%) | 0 ( 0.0%) |
| (col %) | N = 244 | N = 232 |
| TIBIA caliper |  |  |
| Difficulties | 156 (63.9%) | 45 (19.4%) |
| Impossible | 0 ( 0.0%) | 0 ( 0.0%) |
| No difficulties | 88 (36.1%) | 187 (80.6%) |
| Missing data | 0 ( 0.0%) | 0 ( 0.0%) |
| (col %) | N = 244 | N = 232 |
| Knee-Heel tape measure |  |  |
| Difficulties | 7 ( 2.9%) | 15 ( 6.5%) |
| Impossible | 0 ( 0.0%) | 0 ( 0.0%) |
| No difficulties | 237 (97.1%) | 217 (93.5%) |
| Missing data | 0 ( 0.0%) | 0 ( 0.0%) |
| (col %) | N = 244 | N = 232 |
| Knee-Heel caliper |  |  |
| Difficulties | 16 ( 6.6%) | 19 ( 8.2%) |
| Impossible | 0 ( 0.0%) | 0 ( 0.0%) |
| No difficulties | 228 (93.4%) | 213 (91.8%) |
| Missing data | 0 ( 0.0%) | 0 ( 0.0%) |
| (col %) | N = 244 | N = 232 |
| ULNA tape measure |  |  |
| Difficulties | 71 (29.2%) | 19 ( 8.2%) |
| Impossible | 0 ( 0.0%) | 1 ( 0.4%) |
| No difficulties | 172 (70.8%) | 212 (91.4%) |
| Missing data | 0 ( 0.0%) | 0 ( 0.0%) |
| (col %) | N = 243 | N = 232 |
| ULNA caliper |  |  |
| Difficulties | 129 (53.1%) | 28 (12.1%) |
| Impossible | 1 ( 0.4%) | 1 ( 0.4%) |
| No difficulties | 113 (46.5%) | 203 (87.5%) |
| Missing data | 0 ( 0.0%) | 0 ( 0.0%) |
| (col %) | N = 243 | N = 232 |
| Half of the arm span |  |  |
| Difficulties | 64 (26.3%) | 26 (11.2%) |
| Impossible | 2 ( 0.8%) | 3 ( 1.3%) |
| No difficulties | 177 (72.8%) | 203 (87.5%) |
| Missing data | 0 ( 0.0%) | 0 ( 0.0%) |
| (col %) | N = 243 | N = 232 |
| Body segment head |  |  |
| Difficulties | 26 (10.7%) | 34 (14.7%) |
| Impossible | 0 ( 0.0%) | 0 ( 0.0%) |
| No difficulties | 218 (89.3%) | 198 (85.3%) |
| Missing data | 0 ( 0.0%) | 0 ( 0.0%) |
| (col %) | N = 244 | N = 232 |
| Body segment trunk |  |  |
| Difficulties | 116 (47.5%) | 140 (60.3%) |
| Impossible | 0 ( 0.0%) | 0 ( 0.0%) |
| No difficulties | 128 (52.5%) | 92 (39.7%) |
| Missing data | 0 ( 0.0%) | 0 ( 0.0%) |
| (col %) | N = 244 | N = 232 |
| Body segment lower limb |  |  |
| Difficulties | 119 (48.8%) | 143 (61.6%) |
| Impossible | 0 ( 0.0%) | 0 ( 0.0%) |
| No difficulties | 125 (51.2%) | 89 (38.4%) |
| Missing data | 0 ( 0.0%) | 0 ( 0.0%) |
| (col %) | N = 244 | N = 232 |
| Alongside the body tape measure |  |  |
| Difficulties | 29 (11.9%) | 62 (26.7%) |
| Impossible | 2 ( 0.8%) | 3 ( 1.3%) |
| No difficulties | 209 (85.7%) | 158 (68.1%) |
| Missing data | 4 ( 1.6%) | 9 ( 3.9%) |
| (col %) | N = 244 | N = 232 |
| Length board |  |  |
| Difficulties | 30 (12.3%) |  |
| Impossible | 0 ( 0.0%) |  |
| No difficulties | 206 (84.8%) |  |
| Missing data | 7 ( 2.9%) |  |
| (col %) | N = 243 |  |

Supplemental table 14: Difficulties. Number of patients for whom difficulties were encountered during measurements

| **Age group (years)** | **<2** | **≥2** |
| --- | --- | --- |
| Positioning difficulty |  |  |
| TIBIA tape measure |  |  |
| No | 241 (98.8%) | 224 (96.6%) |
| Yes | 3 ( 1.2%) | 8 ( 3.4%) |
| (col %) | N = 244 | N = 232 |
| TIBIA caliper |  |  |
| No | 239 (98.0%) | 224 (96.6%) |
| Yes | 5 ( 2.0%) | 8 ( 3.4%) |
| (col %) | N = 244 | N = 232 |
| Knee-Heel tape measure |  |  |
| No | 242 (99.2%) | 221 (95.3%) |
| Yes | 2 ( 0.8%) | 11 ( 4.7%) |
| (col %) | N = 244 | N = 232 |
| Knee-Heel caliper |  |  |
| No | 241 (98.8%) | 221 (95.3%) |
| Yes | 3 ( 1.2%) | 11 ( 4.7%) |
| (col %) | N = 244 | N = 232 |
| ULNA tape measure |  |  |
| No | 241 (99.2%) | 228 (98.3%) |
| Yes | 2 ( 0.8%) | 4 ( 1.7%) |
| (col %) | N = 243 | N = 232 |
| ULNA caliper |  |  |
| No | 240 (98.8%) | 228 (98.3%) |
| Yes | 3 ( 1.2%) | 4 ( 1.7%) |
| (col %) | N = 243 | N = 232 |
| Half of the arm span |  |  |
| No | 198 (81.5%) | 214 (92.2%) |
| Yes | 45 (18.5%) | 18 ( 7.8%) |
| (col %) | N = 243 | N = 232 |
| Body segment head |  |  |
| No | 231 (94.7%) | 215 (92.7%) |
| Yes | 13 ( 5.3%) | 17 ( 7.3%) |
| (col %) | N = 244 | N = 232 |
| Body segment trunk |  |  |
| No | 239 (98.0%) | 228 (98.3%) |
| Yes | 5 ( 2.0%) | 4 ( 1.7%) |
| (col %) | N = 244 | N = 232 |
| Body segment lower limb |  |  |
| No | 238 (97.5%) | 226 (97.4%) |
| Yes | 6 ( 2.5%) | 6 ( 2.6%) |
| (col %) | N = 244 | N = 232 |
| Alongside the body tape measure |  |  |
| No | 232 (97.5%) | 198 (89.6%) |
| Yes | 6 ( 2.5%) | 23 (10.4%) |
| (col %) | N = 238 | N = 221 |
| Length board |  |  |
| No | 227 (96.2%) |  |
| Yes | 9 ( 3.8%) |  |
| (col %) | N = 236 |  |
| Indwelling device being an obstacle to measurements |  |  |
| TIBIA tape measure |  |  |
| No | 244 (100.0%) | 232 (100.0%) |
| Yes | 0 ( 0.0%) | 0 ( 0.0%) |
| (col %) | N = 244 | N = 232 |
| TIBIA caliper |  |  |
| No | 244 (100.0%) | 229 (98.7%) |
| Yes | 0 ( 0.0%) | 3 ( 1.3%) |
| (col %) | N = 244 | N = 232 |
| Knee-Heel tape measure |  |  |
| No | 244 (100.0%) | 232 (100.0%) |
| Yes | 0 ( 0.0%) | 0 ( 0.0%) |
| (col %) | N = 244 | N = 232 |
| Knee-Heel caliper |  |  |
| No | 244 (100.0%) | 231 (99.6%) |
| Yes | 0 ( 0.0%) | 1 ( 0.4%) |
| (col %) | N = 244 | N = 232 |
| ULNA tape measure |  |  |
| No | 240 (98.8%) | 228 (98.3%) |
| Yes | 3 ( 1.2%) | 4 ( 1.7%) |
| (col %) | N = 243 | N = 232 |
| ULNA caliper |  |  |
| No | 240 (98.8%) | 227 (97.8%) |
| Yes | 3 ( 1.2%) | 5 ( 2.2%) |
| (col %) | N = 243 | N = 232 |
| Half of the arm span |  |  |
| No | 238 (97.9%) | 228 (98.3%) |
| Yes | 5 ( 2.1%) | 4 ( 1.7%) |
| (col %) | N = 243 | N = 232 |
| Body segment head |  |  |
| No | 243 (99.6%) | 225 (97.0%) |
| Yes | 1 ( 0.4%) | 7 ( 3.0%) |
| (col %) | N = 244 | N = 232 |
| Body segment trunk |  |  |
| No | 242 (99.2%) | 228 (98.3%) |
| Yes | 2 ( 0.8%) | 4 ( 1.7%) |
| (col %) | N = 244 | N = 232 |
| Body segment lower limb |  |  |
| No | 243 (99.6%) | 228 (98.3%) |
| Yes | 1 ( 0.4%) | 4 ( 1.7%) |
| (col %) | N = 244 | N = 232 |
| Alongside the body tape measure |  |  |
| No | 236 (99.2%) | 213 (96.4%) |
| Yes | 2 ( 0.8%) | 8 ( 3.6%) |
| (col %) | N = 238 | N = 221 |
| Length board |  |  |
| No | 232 (98.3%) |  |
| Yes | 4 ( 1.7%) |  |
| (col %) | N = 236 |  |
| Difficulty to locate body segments |  |  |
| TIBIA tape measure |  |  |
| No | 118 (48.4%) | 201 (86.6%) |
| Yes | 126 (51.6%) | 31 (13.4%) |
| (col %) | N = 244 | N = 232 |
| TIBIA caliper |  |  |
| No | 116 (47.5%) | 201 (86.6%) |
| Yes | 128 (52.5%) | 31 (13.4%) |
| (col %) | N = 244 | N = 232 |
| Knee-Heel tape measure |  |  |
| No | 242 (99.2%) | 232 (100.0%) |
| Yes | 2 ( 0.8%) | 0 ( 0.0%) |
| (col %) | N = 244 | N = 232 |
| Knee-Heel caliper |  |  |
| No | 241 (98.8%) | 232 (100.0%) |
| Yes | 3 ( 1.2%) | 0 ( 0.0%) |
| (col %) | N = 244 | N = 232 |
| ULNA tape measure |  |  |
| No | 177 (72.8%) | 220 (94.8%) |
| Yes | 66 (27.2%) | 12 ( 5.2%) |
| (col %) | N = 243 | N = 232 |
| ULNA caliper |  |  |
| No | 173 (71.2%) | 218 (94.0%) |
| Yes | 70 (28.8%) | 14 ( 6.0%) |
| (col %) | N = 243 | N = 232 |
| Half of the arm span |  |  |
| No | 234 (96.3%) | 228 (98.3%) |
| Yes | 9 ( 3.7%) | 4 ( 1.7%) |
| (col %) | N = 243 | N = 232 |
| Body segment head |  |  |
| No | 237 (97.1%) | 219 (94.4%) |
| Yes | 7 ( 2.9%) | 13 ( 5.6%) |
| (col %) | N = 244 | N = 232 |
| Body segment trunk |  |  |
| No | 137 (56.1%) | 103 (44.4%) |
| Yes | 107 (43.9%) | 129 (55.6%) |
| (col %) | N = 244 | N = 232 |
| Body segment lower limb |  |  |
| No | 135 (55.3%) | 101 (43.5%) |
| Yes | 109 (44.7%) | 131 (56.5%) |
| (col %) | N = 244 | N = 232 |
| Alongside the body tape measure |  |  |
| No | 237 (99.6%) | 219 (99.1%) |
| Yes | 1 ( 0.4%) | 2 ( 0.9%) |
| (col %) | N = 238 | N = 221 |
| Length board |  |  |
| No | 235 (99.6%) |  |
| Yes | 1 ( 0.4%) |  |
| (col %) | N = 236 |  |
| Difficulty to maintain child’s position during measurements |  |  |
| TIBIA tape measure |  |  |
| No | 237 (97.1%) | 228 (98.3%) |
| Yes | 7 ( 2.9%) | 4 ( 1.7%) |
| (col %) | N = 244 | N = 232 |
| TIBIA caliper |  |  |
| No | 228 (93.4%) | 225 (97.0%) |
| Yes | 16 ( 6.6%) | 7 ( 3.0%) |
| (col %) | N = 244 | N = 232 |
| Knee-Heel tape measure |  |  |
| No | 240 (98.4%) | 227 (97.8%) |
| Yes | 4 ( 1.6%) | 5 ( 2.2%) |
| (col %) | N = 244 | N = 232 |
| Knee-Heel caliper |  |  |
| No | 236 (96.7%) | 225 (97.0%) |
| Yes | 8 ( 3.3%) | 7 ( 3.0%) |
| (col %) | N = 244 | N = 232 |
| ULNA tape measure |  |  |
| No | 235 (96.7%) | 230 (99.1%) |
| Yes | 8 ( 3.3%) | 2 ( 0.9%) |
| (col %) | N = 243 | N = 232 |
| ULNA caliper |  |  |
| No | 232 (95.5%) | 227 (97.8%) |
| Yes | 11 ( 4.5%) | 5 ( 2.2%) |
| (col %) | N = 243 | N = 232 |
| Half of the arm span |  |  |
| No | 204 (84.0%) | 219 (94.4%) |
| Yes | 39 (16.0%) | 13 ( 5.6%) |
| (col %) | N = 243 | N = 232 |
| Body segment head |  |  |
| No | 237 (97.1%) | 228 (98.3%) |
| Yes | 7 ( 2.9%) | 4 ( 1.7%) |
| (col %) | N = 244 | N = 232 |
| Body segment trunk |  |  |
| No | 236 (96.7%) | 231 (99.6%) |
| Yes | 8 ( 3.3%) | 1 ( 0.4%) |
| (col %) | N = 244 | N = 232 |
| Body segment lower limb |  |  |
| No | 230 (94.3%) | 226 (97.4%) |
| Yes | 14 ( 5.7%) | 6 ( 2.6%) |
| (col %) | N = 244 | N = 232 |
| Alongside the body tape measure |  |  |
| No | 217 (91.2%) | 213 (96.4%) |
| Yes | 21 ( 8.8%) | 8 ( 3.6%) |
| (col %) | N = 238 | N = 221 |
| Length board |  |  |
| No | 215 (91.1%) |  |
| Yes | 21 ( 8.9%) |  |
| (col %) | N = 236 |  |
| Difficulties to take measures |  |  |
| TIBIA tape measure |  |  |
| No | 238 (97.5%) | 231 (99.6%) |
| Yes | 6 ( 2.5%) | 1 ( 0.4%) |
| (col %) | N = 244 | N = 232 |
| TIBIA caliper |  |  |
| No | 138 (56.6%) | 217 (93.5%) |
| Yes | 106 (43.4%) | 15 ( 6.5%) |
| (col %) | N = 244 | N = 232 |
| Knee-Heel tape measure |  |  |
| No | 241 (98.8%) | 229 (98.7%) |
| Yes | 3 ( 1.2%) | 3 ( 1.3%) |
| (col %) | N = 244 | N = 232 |
| Knee-Heel caliper |  |  |
| No | 237 (97.1%) | 229 (98.7%) |
| Yes | 7 ( 2.9%) | 3 ( 1.3%) |
| (col %) | N = 244 | N = 232 |
| ULNA tape measure |  |  |
| No | 238 (97.9%) | 231 (99.6%) |
| Yes | 5 ( 2.1%) | 1 ( 0.4%) |
| (col %) | N = 243 | N = 232 |
| ULNA caliper |  |  |
| No | 145 (59.7%) | 218 (94.0%) |
| Yes | 98 (40.3%) | 14 ( 6.0%) |
| (col %) | N = 243 | N = 232 |
| Half of the arm span |  |  |
| No | 232 (95.5%) | 227 (97.8%) |
| Yes | 11 ( 4.5%) | 5 ( 2.2%) |
| (col %) | N = 243 | N = 232 |
| Body segment head |  |  |
| No | 240 (98.4%) | 228 (98.3%) |
| Yes | 4 ( 1.6%) | 4 ( 1.7%) |
| (col %) | N = 244 | N = 232 |
| Body segment trunk |  |  |
| No | 238 (97.5%) | 220 (94.8%) |
| Yes | 6 ( 2.5%) | 12 ( 5.2%) |
| (col %) | N = 244 | N = 232 |
| Body segment lower limb |  |  |
| No | 236 (96.7%) | 220 (94.8%) |
| Yes | 8 ( 3.3%) | 12 ( 5.2%) |
| (col %) | N = 244 | N = 232 |
| Alongside the body tape measure |  |  |
| No | 230 (96.6%) | 181 (81.9%) |
| Yes | 8 ( 3.4%) | 40 (18.1%) |
| (col %) | N = 238 | N = 221 |
| Length board |  |  |
| No | 234 (99.2%) |  |
| Yes | 2 ( 0.8%) |  |
| (col %) | N = 236 |  |
| Difficulty to read the measure |  |  |
| TIBIA tape measure |  |  |
| No | 244 (100.0%) | 232 (100.0%) |
| Yes | 0 ( 0.0%) | 0 ( 0.0%) |
| (col %) | N = 244 | N = 232 |
| TIBIA caliper |  |  |
| No | 240 (98.4%) | 232 (100.0%) |
| Yes | 4 ( 1.6%) | 0 ( 0.0%) |
| (col %) | N = 244 | N = 232 |
| Knee-Heel tape measure |  |  |
| No | 243 (99.6%) | 232 (100.0%) |
| Yes | 1 ( 0.4%) | 0 ( 0.0%) |
| (col %) | N = 244 | N = 232 |
| Knee-Heel caliper |  |  |
| No | 242 (99.2%) | 231 (99.6%) |
| Yes | 2 ( 0.8%) | 1 ( 0.4%) |
| (col %) | N = 244 | N = 232 |
| ULNA tape measure |  |  |
| No | 243 (100.0%) | 232 (100.0%) |
| Yes | 0 ( 0.0%) | 0 ( 0.0%) |
| (col %) | N = 243 | N = 232 |
| ULNA caliper |  |  |
| No | 239 (98.4%) | 231 (99.6%) |
| Yes | 4 ( 1.6%) | 1 ( 0.4%) |
| (col %) | N = 243 | N = 232 |
| Half of the arm span |  |  |
| No | 243 (100.0%) | 231 (99.6%) |
| Yes | 0 ( 0.0%) | 1 ( 0.4%) |
| (col %) | N = 243 | N = 232 |
| Body segment head |  |  |
| No | 243 (99.6%) | 232 (100.0%) |
| Yes | 1 ( 0.4%) | 0 ( 0.0%) |
| (col %) | N = 244 | N = 232 |
| Body segment trunk |  |  |
| No | 243 (99.6%) | 232 (100.0%) |
| Yes | 1 ( 0.4%) | 0 ( 0.0%) |
| (col %) | N = 244 | N = 232 |
| Body segment lower limb |  |  |
| No | 243 (99.6%) | 232 (100.0%) |
| Yes | 1 ( 0.4%) | 0 ( 0.0%) |
| (col %) | N = 244 | N = 232 |
| Alongside the body tape measure |  |  |
| No | 238 (100.0%) | 215 (97.3%) |
| Yes | 0 ( 0.0%) | 6 ( 2.7%) |
| (col %) | N = 238 | N = 221 |
| Length board |  |  |
| No | 236 (100.0%) |  |
| Yes | 0 ( 0.0%) |  |
| (col %) | N = 236 |  |

Supplemental table 15: Safety. Number of patients for whom safety issues were encountered during measurements

| **Age group (years)** | **<2** | **≥2** |
| --- | --- | --- |
| Safety assessment |  |  |
| TIBIA tape measure |  |  |
| Danger | 0 ( 0.0%) | 0 ( 0.0%) |
| No danger | 244 (100.0%) | 232 (100.0%) |
| Missing data | 0 ( 0.0%) | 0 ( 0.0%) |
| (col %) | N = 244 | N = 232 |
| TIBIA caliper |  |  |
| Danger | 3 ( 1.2%) | 0 ( 0.0%) |
| No danger | 241 (98.8%) | 232 (100.0%) |
| Missing data | 0 ( 0.0%) | 0 ( 0.0%) |
| (col %) | N = 244 | N = 232 |
| Knee-Heel tape measure |  |  |
| Danger | 0 ( 0.0%) | 0 ( 0.0%) |
| No danger | 244 (100.0%) | 232 (100.0%) |
| Missing data | 0 ( 0.0%) | 0 ( 0.0%) |
| (col %) | N = 244 | N = 232 |
| Knee-Heel caliper |  |  |
| Danger | 3 ( 1.2%) | 0 ( 0.0%) |
| No danger | 241 (98.8%) | 232 (100.0%) |
| Missing data | 0 ( 0.0%) | 0 ( 0.0%) |
| (col %) | N = 244 | N = 232 |
| ULNA tape measure |  |  |
| Danger | 0 ( 0.0%) | 0 ( 0.0%) |
| No danger | 244 (100.0%) | 232 (100.0%) |
| Missing data | 0 ( 0.0%) | 0 ( 0.0%) |
| (col %) | N = 244 | N = 232 |
| ULNA caliper |  |  |
| Danger | 2 ( 0.8%) | 1 ( 0.4%) |
| No danger | 242 (99.2%) | 231 (99.6%) |
| Missing data | 0 ( 0.0%) | 0 ( 0.0%) |
| (col %) | N = 244 | N = 232 |
| Half of the arm span |  |  |
| Danger | 2 ( 0.8%) | 0 ( 0.0%) |
| No danger | 242 (99.2%) | 232 (100.0%) |
| Missing data | 0 ( 0.0%) | 0 ( 0.0%) |
| (col %) | N = 244 | N = 232 |
| Body segment head |  |  |
| Danger | 0 ( 0.0%) | 0 ( 0.0%) |
| No danger | 244 (100.0%) | 232 (100.0%) |
| Missing data | 0 ( 0.0%) | 0 ( 0.0%) |
| (col %) | N = 244 | N = 232 |
| Body segment trunk |  |  |
| Danger | 0 ( 0.0%) | 0 ( 0.0%) |
| No danger | 244 (100.0%) | 232 (100.0%) |
| Missing data | 0 ( 0.0%) | 0 ( 0.0%) |
| (col %) | N = 244 | N = 232 |
| Body segment lower limb |  |  |
| Danger | 1 ( 0.4%) | 0 ( 0.0%) |
| No danger | 243 (99.6%) | 232 (100.0%) |
| Missing data | 0 ( 0.0%) | 0 ( 0.0%) |
| (col %) | N = 244 | N = 232 |
| Alongside the body tape measure |  |  |
| Danger | 1 ( 0.4%) | 0 ( 0.0%) |
| No danger | 239 (98.0%) | 223 (96.1%) |
| Missing data | 4 ( 1.6%) | 9 ( 3.9%) |
| (col %) | N = 244 | N = 232 |
| Length board |  |  |
| Danger | 0 ( 0.0%) |  |
| No danger | 236 (97.1%) |  |
| Missing data | 7 ( 2.9%) |  |
| (col %) | N = 243 |  |

Supplemental table 16 : Inter rater reliability. Intraclass correlation coefficient (ICC) and its 95% confidence interval in age subgroups below and above 2 years of age

| **Methods** | **ICC** | **95% CI** |
| --- | --- | --- |
| TIBIA tape measure |  |  |
| <2 | 0.92 | [0.85 ; 0.95] |
| ≥2 | 0.99 | [0.98 ; 0.99] |
| TIBIA caliper |  |  |
| <2 | 0.93 | [0.88 ; 0.96] |
| ≥2 | 0.98 | [0.97 ; 0.99] |
| Knee-Heel tape measure Gauld |  |  |
| <2 | 0.96 | [0.93 ; 0.98] |
| ≥2 | 0.99 | [0.99 ; 1] |
| Knee-Heel caliper Gauld |  |  |
| <2 | 0.97 | [0.95 ; 0.99] |
| ≥2 | 1 | [0.99 ; 1] |
| Knee-Heel tape measure Chumlea |  |  |
| <2 | 0.96 | [0.93 ; 0.98] |
| ≥2 | 0.99 | [0.99 ; 1] |
| Knee-Heel caliper Chumlea |  |  |
| <2 | 0.97 | [0.95 ; 0.99] |
| ≥2 | 1 | [0.99 ; 1] |
| ULNA tape measure |  |  |
| <2 | 0.96 | [0.94 ; 0.98] |
| ≥2 | 0.99 | [0.98 ; 0.99] |
| ULNA caliper |  |  |
| <2 | 0.95 | [0.91 ; 0.97] |
| ≥2 | 0.98 | [0.97 ; 0.99] |
| Half of the arm span |  |  |
| <2 | 0.96 | [0.93 ; 0.98] |
| ≥2 | 1 | [0.99 ; 1] |
| Body segment head |  |  |
| <2 | 0.78 | [0.63 ; 0.87] |
| ≥2 | 0.75 | [0.62 ; 0.83] |
| Body segment trunk |  |  |
| <2 | 0.73 | [0.56 ; 0.84] |
| ≥2 | 0.78 | [0.67 ; 0.86] |
| Body segment lower limb |  |  |
| <2 | 0.92 | [0.87 ; 0.96] |
| ≥2 | 0.97 | [0.96 ; 0.98] |
| Sum of body segments |  |  |
| <2 | 0.96 | [0.93 ; 0.98] |
| ≥2 | 0.98 | [0.96 ; 0.99] |
| Alongside the body tape measure |  |  |
| <2 | 0.99 | [0.98 ; 0.99] |
| ≥2 | 0.99 | [0.99 ; 1] |
| Length board |  |  |
| <2 | 0.99 | [0.99 ; 1] |
| ≥2 | - | - |

Supplemental table 17: Inter rater reliability. Difference between the two measurements, according to the age-group

| **Age group (years)** | **<2** | **≥2** |
| --- | --- | --- |
| Difference between the 2 measurements (cm) |  |  |
| TIBIA tape measure |  |  |
| Mean (SD) | 0.23 ( 0.98) | 0.26 ( 1.47) |
| Median | 0.40 | 0.00 |
| Q1-Q3 | -0.25 - 1.00 | -0.50 - 1.00 |
| Min-Max | -2.70 - 2.00 | -4.50 - 4.90 |
| N | 47 | 72 |
| TIBIA caliper |  |  |
| Mean (SD) | 0.06 ( 0.91) | 0.38 ( 1.71) |
| Median | 0.00 | 0.25 |
| Q1-Q3 | -0.40 - 0.60 | -0.33 - 1.05 |
| Min-Max | -1.80 - 2.50 | -5.30 - 5.30 |
| N | 47 | 72 |
| Knee-Heel tape measure Gauld |  |  |
| Mean (SD) | -0.17 ( 0.98) | 0.01 ( 1.20) |
| Median | 0.00 | 0.00 |
| Q1-Q3 | -0.50 - 0.00 | -1.00 - 0.50 |
| Min-Max | -2.50 - 3.60 | -2.50 - 4.20 |
| N | 47 | 72 |
| Knee-Heel caliper Gauld |  |  |
| Mean (SD) | 0.12 ( 0.77) | 0.17 ( 1.00) |
| Median | 0.10 | 0.05 |
| Q1-Q3 | -0.25 - 0.45 | -0.50 - 0.53 |
| Min-Max | -2.30 - 2.00 | -2.00 - 4.00 |
| N | 47 | 72 |
| Knee-Heel tape measure Chumlea |  |  |
| Mean (SD) | -0.17 ( 0.98) | 0.01 ( 1.20) |
| Median | 0.00 | 0.00 |
| Q1-Q3 | -0.50 - 0.00 | -1.00 - 0.50 |
| Min-Max | -2.50 - 3.60 | -2.50 - 4.20 |
| N | 47 | 72 |
| Knee-Heel caliper Chumlea |  |  |
| Mean (SD) | 0.12 ( 0.77) | 0.17 ( 1.00) |
| Median | 0.10 | 0.05 |
| Q1-Q3 | -0.25 - 0.45 | -0.50 - 0.53 |
| Min-Max | -2.30 - 2.00 | -2.00 - 4.00 |
| N | 47 | 72 |
| ULNA tape measure |  |  |
| Mean (SD) | 0.27 ( 0.48) | 0.19 ( 0.85) |
| Median | 0.30 | 0.00 |
| Q1-Q3 | 0.00 - 0.50 | -0.45 - 0.50 |
| Min-Max | -0.70 - 1.50 | -2.00 - 3.20 |
| N | 47 | 71 |
| ULNA caliper |  |  |
| Mean (SD) | 0.15 ( 0.56) | 0.17 ( 0.97) |
| Median | 0.20 | 0.10 |
| Q1-Q3 | -0.10 - 0.50 | -0.35 - 0.50 |
| Min-Max | -1.30 - 1.20 | -1.60 - 5.40 |
| N | 47 | 71 |
| Half of the arm span |  |  |
| Mean (SD) | 0.07 ( 1.61) | -0.25 ( 1.53) |
| Median | 0.00 | 0.00 |
| Q1-Q3 | -1.00 - 0.70 | -1.00 - 0.50 |
| Min-Max | -3.80 - 3.50 | -3.50 - 3.00 |
| N | 47 | 71 |
| Body segment head |  |  |
| Mean (SD) | -0.02 ( 1.31) | 0.66 ( 2.31) |
| Median | 0.00 | 0.75 |
| Q1-Q3 | -1.00 - 0.75 | -0.50 - 2.00 |
| Min-Max | -2.10 - 3.50 | -4.00 - 10.00 |
| N | 47 | 72 |
| Body segment trunk |  |  |
| Mean (SD) | 0.08 ( 2.74) | 0.11 ( 6.75) |
| Median | 0.00 | 0.00 |
| Q1-Q3 | -1.50 - 1.75 | -3.00 - 2.00 |
| Min-Max | -10.50 - 5.50 | -15.50 - 28.20 |
| N | 47 | 72 |
| Body segment head lower limb |  |  |
| Mean (SD) | -0.44 ( 2.49) | 0.70 ( 4.48) |
| Median | 0.00 | 0.55 |
| Q1-Q3 | -1.75 - 0.90 | -1.50 - 3.00 |
| Min-Max | -6.50 - 4.30 | -23.00 - 13.00 |
| N | 47 | 72 |
| Sum of body segments |  |  |
| Mean (SD) | -0.38 ( 2.99) | 1.48 ( 6.36) |
| Median | 0.00 | 1.50 |
| Q1-Q3 | -1.60 - 1.85 | -1.00 - 3.00 |
| Min-Max | -10.00 - 5.00 | -21.50 - 25.20 |
| N | 47 | 72 |
| Alongside the body tape measure |  |  |
| Mean (SD) | -0.47 ( 1.42) | -1.11 ( 3.29) |
| Median | -0.50 | -1.00 |
| Q1-Q3 | -1.07 - 0.50 | -2.00 - 1.00 |
| Min-Max | -4.00 - 2.50 | -14.00 - 5.00 |
| N | 44 | 64 |
| Length board |  |  |
| Mean (SD) | 0.05 ( 1.12) |  |
| Median | 0.00 |  |
| Q1-Q3 | -0.50 - 0.50 |  |
| Min-Max | -2.40 - 4.50 |  |
| N | 44 |  |
