# Supplementary material for: Accurate height and length estimation in hospitalized children not fulfilling WHO criteria for standard measurement: a multicenter prospective study
Source: Eur J Pediatr. 2024 Jul 25;183(10):4275–86. doi: 10.1007/s00431-024-05692-3 (PMC11413069; doi:10.1007/s00431-024-05692-3)
Supplement: Supplementary file 4 — Supplementary file4 (DOCX 28 KB) [file 431_2024_5692_MOESM4_ESM.docx]

Supplemental table 18: Bias, percentage of absolute value of the relative error, number of difficulties or safety issues and intraclass correlation coefficients of each extrapolation or estimation of height or length methods in children < 2years

| **Length extrapolation or estimation method** | **Mean bias (cm)** | **Mean bias (cm) 95% CI** | **Limits of agreement** | **% of the relative error <3.5%** | **CCC** | **95% CI** | **Difficulties**  **(N and %)** | **Safety issue**  **(N and %)** | **Interrater reliability**  **ICC**  **N=47** | **Interrater reliability**  **ICC 95% CI**  **N=47** |
| --- | --- | --- | --- | --- | --- | --- | --- | --- | --- | --- |
| Tibia tape measure | 3.61 | [2.95 ; 4.27] | [-6.57 ; 13.78] | 29.71 | 0.78 | [0.74 ; 0.81] | 131 (53.7%) | 0 (0.0%) | 0.92 | [0.85 ; 0.95] |
| Tibia caliper | 3.17 | [2.52 ; 3.82] | [-6.84 ; 13.18] | 32.64 | 0.79 | [0.75 ; 0.82] | 156 (63.9%) | 3 (1.2%) | 0.93 | [0.88 ; 0.96] |
| Knee-Heel tape measure Gauld | -0.4 | [-0.88 ; 0.08] | [-7.86 ; 7.07] | 51.88 | 0.92 | [0.9 ; 0.94] | 7 (2.9%) | 0 (0.0%) | 0.96 | [0.93 ; 0.98] |
| Knee-Heel caliper Gauld | -2.11 | [-2.58 ; -1.64] | [-9.35 ; 5.13] | 39.75 | 0.91 | [0.88 ; 0.92] | 16 (6.6%) | 3 (1.2%) | 0.97 | [0.95 ; 0.99] |
| Knee-Heel tape meas. Chumlea | 14.74 | [14.13 ; 15.34] | [5.38 ; 24.1] | 2.09 | 0.37 | [0.32 ; 0.41] | 7 (2.9%) | 0 (0.0%) | 0.96 | [0.93 ; 0.98] |
| Knee-Heel caliper Chumlea | 13.21 | [12.61 ; 13.8] | [3.96 ; 22.45] | 2.09 | 0.42 | [0.37 ; 0.46] | 16 (6.6%) | 3 (1.2%) | 0.97 | [0.95 ; 0.99] |
| Ulna tape measure | 8.38 | [7.76 ; 8.99] | [-1.14 ; 17.89] | 8.37 | 0.62 | [0.56 ; 0.66] | 71 (29.2%) | 0 (0.0%) | 0.96 | [0.94 ; 0.98] |
| Ulna caliper | 8.15 | [7.51 ; 8.8] | [-1.79 ; 18.09] | 8.82 | 0.61 | [0.56 ; 0.66] | 129 (53.1%) | 2 (0.8%) | 0.95 | [0.91 ; 0.97] |
| Half of the arm span | 8.85 | [8.09 ; 9.6] | [-2.77 ; 20.46] | 9.7 | 0.58 | [0.52 ; 0.63] | 64 (26.3%) | 2 (0.8%) | 0.96 | [0.93 ; 0.98] |
| Sum of body segments | 0.53 | [0.16 ; 0.89] | [-5.09 ; 6.15] | 64.85 | 0.96 | [0.95 ; 0.97] |  |  | 0.96 | [0.93 ; 0.98] |
| Head |  |  |  |  |  |  | 26 (10.7%) | 0 (0.0%) | 0.78 | [0.63 ; 0.87] |
| Trunk |  |  |  |  |  |  | 116 (47.5%) | 0 (0.0%) | 0.73 | [0.56 ; 0.84] |
| Lower limb |  |  |  |  |  |  | 119 (48.8%) | 1 (0.4%) | 0.92 | [0.87 ; 0.96] |
| Alongside the body tape measure | -0.02 | [-0.31 ; 0.26] | [-4.38 ; 4.33] | 77.68 | 0.98 | [0.97 ; 0.98] | 29 (11.9%) | 1 (0.4%) | 0.99 | [0.98 ; 0.99] |
| Length board | -0.26 | [-0.48 ; -0.04] | [-3.63 ; 3.1] | 86.21 | 0.99 | [0.98 ; 0.99] | 30 (12.3%) | 0 (0.0%) | 0.99 | [0.99 ; 1] |
| Growth chart extrapolation | -0.14 | [-0.36 ; 0.07] | [-3.36 ; 3.08] | 84.44 | 0.99 | [0.98 ; 0.99] |  |  |  |  |
| Weight for age z-score extrapolation | -1.01 | [-1.4 ; -0.61] | [-7.03 ; 5.02] | 51.26 | 0.95 | [0.94 ; 0.96] |  |  |  |  |
| Genetic target extrapolation | 2.03 | [1.49 ; 2.56] | [-6.04 ; 10.09] | 49.34 | 0.90 | [0.88 ; 0.92] |  |  |  |  |
| Parents’ report | -0.72 | [-1.05 ; -0.38] | [-5.29 ; 3.86] | 72.49 | 0.97 | [0.96 ; 0.98] |  |  |  |  |
| Medical file | -1.61 | [-1.94 ; -1.28] | [-6.55 ; 3.33] | 69.43 | 0.96 | [0.95 ; 0.97] |  |  |  |  |

Supplemental table 19: Bias, percentage of absolute value of the relative error, number of difficulties or safety issues and intraclass correlation coefficients of each extrapolation or estimation of height or length methods in children >2years

| Height extrapolation or estimation method | **Mean bias (cm)** | **Mean bias (cm) 95% CI** | **Limits of agreement** | **% of the relative error <3.5%** | **CCC** | **95% CI** | **Difficulties**  **(N and %)** | **Safety issue**  **(N and %)** | **Interrater reliability**  **ICC**  N=72 | **Interrater reliability**  **ICC 95% CI**  N=72 |
| --- | --- | --- | --- | --- | --- | --- | --- | --- | --- | --- |
|  |  |  |  |  |  |  |  |  |  |  |
| Tibia tape measure | -0.53 | [-1.36 ; 0.3] | [-12.93 ; 11.87] | 64.57 | 0.98 | [0.97 ; 0.98] | 37 (15.9%) | 0 (0.0%) | 0.99 | [0.98 ; 0.99] |
| Tibia caliper | -1.42 | [-2.25 ; -0.58] | [-13.93 ; 11.09] | 65.02 | 0.98 | [0.97 ; 0.98] | 45 (19.4%) | 0 (0.0%) | 0.98 | [0.97 ; 0.99] |
| Knee-Heel tape measure Gauld | 0.01 | [-0.63 ; 0.66] | [-9.64 ; 9.66] | 66.82 | 0.99 | [0.98 ; 0.99] | 15 (6.5%) | 0 (0.0%) | 0.99 | [0.99 ; 1] |
| Knee-Heel caliper Gauld | -1.28 | [-2 ; -0.55] | [-12.09 ; 9.53] | 64.13 | 0.98 | [0.98 ; 0.99] | 19 (8.2%) | 0 (0.0%) | 1 | [0.99 ; 1] |
| Knee-Heel tape meas. Chumlea | -1.19 | [-2.11 ; -0.27] | [-14.98 ; 12.6] | 40.81 | 0.96 | [0.96 ; 0.97] | 15 (6.5%) | 0 (0.0%) | 0.99 | [0.99 ; 1] |
| Knee-Heel caliper Chumlea | -2.35 | [-3.3 ; -1.39] | [-16.6 ; 11.91] | 39.91 | 0.96 | [0.95 ; 0.97] | 19 (8.2%) | 0 (0.0%) | 1 | [0.99 ; 1] |
| Ulna tape measure | 1.49 | [0.71 ; 2.28] | [-10.2 ; 13.18] | 59.46 | 0.98 | [0.97 ; 0.98] | 19 (8.2%) | 0 (0.0%) | 0.99 | [0.98 ; 0.99] |
| Ulna caliper | 0.94 | [0.22 ; 1.66] | [-9.83 ; 11.72] | 61.71 | 0.98 | [0.98 ; 0.99] | 28 (12.5%) | 1 (0.4%) | 0.98 | [0.97 ; 0.99] |
| Half of the arm span | 1.34 | [0.78 ; 1.9] | [-6.97 ; 9.66] | 67.27 | 0.99 | [0.98 ; 0.99] | 26 (11.2%) | 0 (0.0%) | 1 | [0.99 ; 1] |
| Sum of body segments | 1.99 | [1.41 ; 2.57] | [-6.7 ; 10.68] | 73.09 | 0.99 | [0.98 ; 0.99] |  |  | 0.98 | [0.96 ; 0.99] |
| Head |  |  |  |  |  |  | 34 (14.7%) | 0 (0.0%) | 0.75 | [0.62 ; 0.83] |
| Trunk |  |  |  |  |  |  | 140 (60.3%) | 0 (0.0%) | 0.78 | [0.67 ; 0.86] |
| Lower limb |  |  |  |  |  |  | 143 (61.6%) | 0 (0.0%) | 0.97 | [0.96 ; 0.98] |
| Alongside the body tape measure | 1.9 | [1.42 ; 2.38] | [-5.03 ; 8.83] | 75.83 | 0.99 | [0.99 ; 0.99] | 62 (26.7%) | 0 (0.0%) | 0.99 | [0.99 ; 1] |
| Growth chart extrapolation | -0.69 | [-1.67 ; 0.28] | [-14.89 ; 13.51] | 90.57 | 0.97 | [0.96 ; 0.98] |  |  |  |  |
| Weight for age z-score extrapolation | 0.47 | [-0.5 ; 1.44] | [-14 ; 14.93] | 58.3 | 0.97 | [0.96 ; 0.98] |  |  |  |  |
| Genetic target extrapolation | 3.17 | [1.45 ; 4.89] | [-21.51 ; 27.85] | 53.88 | 0.90 | [0.88 ; 0.93] |  |  |  |  |
| Parents’ report | -0.42 | [-0.89 ; 0.05] | [-6.72 ; 5.88] | 89.01 | 0.99 | [0.99 ; 1] |  |  |  |  |
| Medical file | -4.89 | [-6.03 ; -3.74] | [-21.62 ; 11.85] | 69.48 | 0.94 | [0.93 ; 0.96] |  |  |  |  |
