# Supplementary material for: Accurate height and length estimation in hospitalized children not fulfilling WHO criteria for standard measurement: a multicenter prospective study
Source: Eur J Pediatr. 2024 Jul 25;183(10):4275–86. doi: 10.1007/s00431-024-05692-3 (PMC11413069; doi:10.1007/s00431-024-05692-3)
Supplement: Supplementary file 5 — Supplementary file5 (DOCX 18 KB) [file 431_2024_5692_MOESM5_ESM.docx]

Supplemental table 20: Percentage of patients with absolute relative error less than 3.5% - Age < 2 years

| **Methods** | **Lyon centers**  **(% [95% CI])** | **Other centers**  **(% [95% CI])** |
| --- | --- | --- |
| Tibia tape | 29.4 [22.6 ; 37.1] | 30.3 [20.2 ; 41.9] |
| Tibia caliper | 31.9 [24.8 ; 39.6] | 34.2 [23.7 ; 46] |
| Knee-heel tape Gauld | 51.5 [43.6 ; 59.4] | 52.6 [40.8 ; 64.2] |
| Knee-heel caliper Gauld | 40.5 [32.9 ; 48.4] | 38.2 [27.2 ; 50] |
| Knee-heel tape Chumlea | 0.6 [0 ; 3.4] | 5.3 [1.5 ; 12.9] |
| Knee-heel caliper Chumlea | 1.2 [0.1 ; 4.4] | 3.9 [0.8 ; 11.1] |
| Ulna tape | 7.4 [3.9 ; 12.5] | 10.5 [4.7 ; 19.7] |
| Ulna caliper | 8 [4.3 ; 13.3] | 10.7 [4.7 ; 19.9] |
| Half of the arm span | 7.5 [3.9 ; 12.7] | 14.5 [7.5 ; 24.4] |
| Sum of body segments | 74.2 [66.8 ; 80.8] | 44.7 [33.3 ; 56.6] |
| Alongside the body tape | 82.8 [76.1 ; 88.3] | 65.7 [53.4 ; 76.7] |
| Length board | 88.3 [82.4 ; 92.8] | 81.2 [69.9 ; 89.6] |
| Growth curves | 91.3 [85.8 ; 95.2] | 67.2 [54.3 ; 78.4] |
| Weight for age z-score extrapolation | 50.9 [43 ; 58.8] | 52 [40.2 ; 63.7] |
| Genetic target height extrapolation | 46.5 [38.5 ; 54.6] | 55.6 [43.4 ; 67.3] |
| Parents' report | 79.1 [71 ; 85.7] | 58.3 [44.9 ; 70.9] |
| Medical file | 75.2 [67.7 ; 81.6] | 55.9 [43.3 ; 67.9] |

Supplemental table 21: Percentage of patients with absolute relative error greater than 3.5% - Age ≥ 2 years

| **Methods** | **Lyon centers**  **(% [95% CI])** | **Other centers**  **(% [95% CI])** |
| --- | --- | --- |
| Tibia tape | 73.4 [65.2 ; 80.5] | 50 [38.9 ; 61.1] |
| Tibia caliper | 71.9 [63.7 ; 79.2] | 53.6 [42.4 ; 64.5] |
| Knee-heel tape Gauld | 74.1 [66 ; 81.2] | 54.8 [43.5 ; 65.7] |
| Knee-heel caliper Gauld | 66.9 [58.4 ; 74.6] | 59.5 [48.3 ; 70.1] |
| Knee-heel tape Chumlea | 35.3 [27.3 ; 43.8] | 50 [38.9 ; 61.1] |
| Knee-heel caliper Chumlea | 40.3 [32.1 ; 48.9] | 39.3 [28.8 ; 50.5] |
| Ulna tape | 65.5 [56.9 ; 73.3] | 49.4 [38.2 ; 60.6] |
| Ulna caliper | 71.2 [62.9 ; 78.6] | 45.8 [34.8 ; 57.1] |
| Half of the arm span | 71 [62.7 ; 78.4] | 61 [49.6 ; 71.6] |
| Sum of body segments | 83.5 [76.2 ; 89.2] | 56 [44.7 ; 66.8] |
| Alongside the body tape | 81.3 [73.8 ; 87.4] | 65.3 [53.1 ; 76.1] |
| Growth curves | 94 [88.6 ; 97.4] | 84.6 [74.7 ; 91.8] |
| Weight for age z-score extrapolation | 61.2 [52.5 ; 69.3] | 53.6 [42.4 ; 64.5] |
| Genetic target height extrapolation | 56.1 [47.2 ; 64.7] | 50 [38.1 ; 61.9] |
| Parents' report | 89.7 [82.6 ; 94.5] | 87.9 [77.5 ; 94.6] |
| Medical file | 73.1 [64.8 ; 80.4] | 63.3 [51.7 ; 73.9] |
